# Molecular structure and modeling studies of azobenzene derivatives containing maleimide groups

**DOI:** 10.1186/2193-1801-2-586

**Published:** 2013-10-31

**Authors:** Corneliu Cojocaru, Anton Airinei, Nicusor Fifere

**Affiliations:** “Petru Poni” Institute of Macromolecular Chemistry, Aleea Grigore Ghica Voda 41A, 700487 Iasi, Romania

**Keywords:** Azobenzene derivatives, Isomers, Molecular orbital calculations, Electronic absorption spectra, HOMO-LUMO

## Abstract

The molecular orbital calculations have been carried out to investigate the structure and stability of (E) / (Z) isomers of some azobenzene derivatives containing maleimide groups. A special attention has been devoted to the compound (E)-1, (E)-1-(4-(phenyldiazenyl)phenyl)-1H-pyrrole-2,5-dione, for which the available crystallographic experimental data have been used to validate the modeling structures computed at the theoretical levels AM1, PM3, RHF/6-31+G(d,p) and B3LYP/6-31+G(d,p). To this end, the discrepancy between experimental and calculated structural parameters has been ascertained in terms of root-mean-square deviation *(RMSD*). The quantum calculations at the level RHF/6-31+G(d,p) yield the most accurate results on (E)-1 structure giving a deviation error from crystallographic data of about 5.00% for bond lengths and 0.97% for interatomic angles. The theoretical electronic absorption spectra of azobenzene derivatives of concern have been computed by means of configuration-interaction method (CI) at the level of semi-empirical Hamiltonians (AM1 and PM3). Likewise, the molecular energy spectra, electrostatic potential and some quantitative structure activity relationship (QSAR) properties of studied molecules have been computed and discussed in the paper.

**Electronic supplementary material:**

The online version of this article (doi:10.1186/2193-1801-2-586) contains supplementary material, which is available to authorized users.

## Introduction

Azobenzene derivatives are molecularly versatile materials capable to provide a response to external stimuli, which allow the control of their photophysical properties. Due to their unique (E) / (Z) / (E) isomerization process, the azobenzene derivatives found a wide applicability in many fields such as photoresponsive materials, optical data storage, surface relief gratings, optoelectronics switches, molecular machines, liquid crystal displays, etc. (Rau 1990a; Kumar & Neckers 1989; Bouas-Laurent & Durr 2001; Natansohn & Rochon 2002; Angiolini et al. 2011; Ebead 2011; Fayet et al. 2010; Hamelmann et al. 2004; Matczyszyn et al. 2003; Kay et al. 2007; Merino & Ribagorda 2012; Wang & Zhang 2012; Delaire & Nakatani 2000; Luftor et al. 2009; Ishow et al. 2006).

It is worth noting that one of the most intriguing aspects of azobenzene compounds is the photo-isomerization of (E) and (Z) isomers and their recovery to the starting (E) isomer. Knowledge of the structure of azobenzene derivative conformers is essential for the understanding the (E) / (Z) / (E) isomerization mechanism (Shaabani & Zahedi 2000; Kurita et al. 2002; Rau 1990b; Diau 2004; Schultz et al. 2003; Satzger et al. 2003; Airinei et al. 2010). It is well-known that the azobenzene compounds exhibit two absorption bands, an intense band located in the UV spectral region assigned to π → π^*^ and a weak band which can be found in the visible region assigned to n → π^*^ electronic transition. Upon UV light (365 nm) irradiation, the energetically more stable (E) isomer can be converted to the non-planar (Z) isomer. The (Z) / (E) backward isomerization process proceeds thermally or with blue light irradiation. The reversible (Z) / (E) photoisomerization of azobenzene moiety represents generally the basis for obtaining azobenzene-based photoresponsive materials (Bouas-Laurent & Durr 2001; Natansohn & Rochon 2002; Rau 1990b; Yager & Barett 2006). Since the applications require the design of the specific azobenzene derivatives with given spectroscopic or photochromic characteristics, the theoretical calculations play an important role for revealing new structures and properties beside the traditional synthesis procedures.

Morley and co-workers (Morley et al. 2004) performed the molecular modeling studies on the photochemical stability of some azobenzene derivatives. The structure and electronic properties of a series of colored azobenzenes containing electron donors and attractors were calculated with both semi-empirical (AM1, PM3) and ab-initio methods. A good agreement was found between the predicted positions of the first and second absorption bands of the azo dyes calculated with AM1/multi-electron configuration interaction method and the experimental values determined in methanol.

A detailed MNDO, AM1 and PM3 study on the stability of isomers of some p,p’-substituted azobenzene derivatives was reported (Shaabani & Zahedi 2000). The results of their computations showed that according to MNDO and PM3 Hamiltonians (E) isomer was the most stable being in agreement with experimental results. However AM1 method suggests that (Z) isomer was more stable, contrary to all experimental data.

Matsuura and collaborators (Matsuura et al. 2008) estimated the absorption maxima of some azobenzene derivatives using the AM1, PM3 and PM5 semi-empirical molecular orbital methods with the configuration interaction singles and random phase approximation calculations. These authors found out that absorption maxima determined by semi-empirical methods were in good correlations with the observed counterpart values.

In this work, semi-empirical methods (AM1 and PM3) have been employed as basic models to study theoretically some azobenzene derivatives containing maleimide moieties and to compare the modeling results with the available experimental data. The ground state and excited state, as well as their electronic structures and optical properties are discussed.

## Methodology

### Modeled compounds

**Figure 1:**
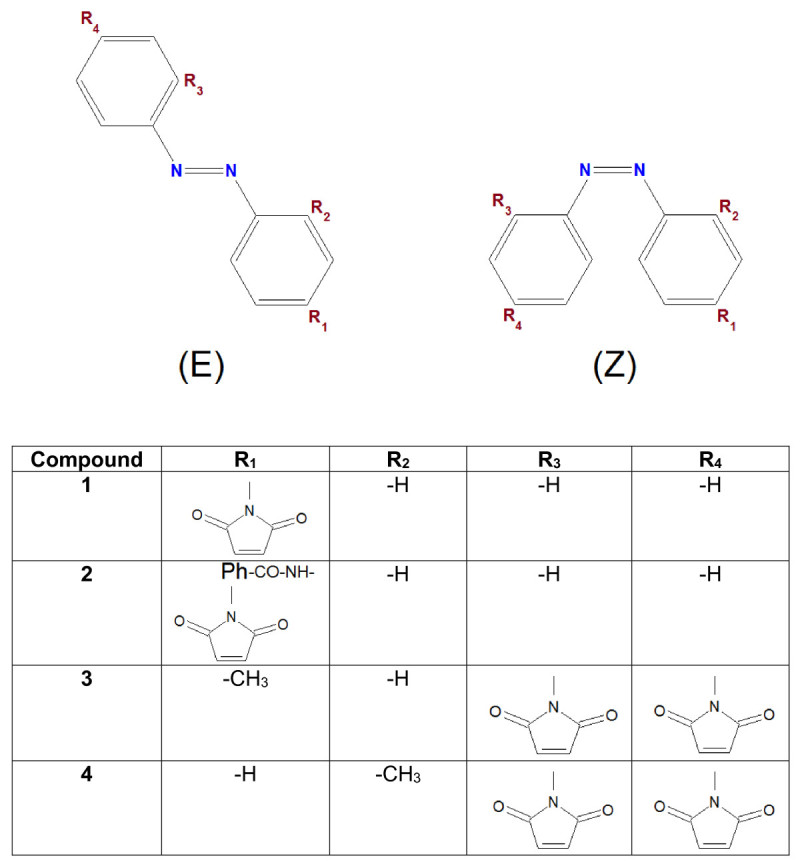
**Chemical structure of azobenzene derivatives considered for molecular modeling.**

The chemical structures of azobenzene derivatives considered for molecular modeling are shown in the Figure 1. Thus, four compounds (1–4) have been considered for modeling, and each one involves two isomers (E) and (Z). The titles of these compounds are listed as follows, (E)-1: (E)-1-(4-(phenyldiazenyl)phenyl)-1H-pyrrole-2,5-dione; (Z)-1: (Z)-1-(4-(phenyldiazenyl)phenyl)-1H-pyrrole-2,5-dione; (E)-2: (E)-4-(2,5-dioxo-2*H*-pyrrol-1(5*H*)-yl)-*N*-(4-(phenyldiazenyl)phenyl) benzamide; (Z)-2: (Z)-4-(2,5-dioxo-2*H*-pyrrol-1(5*H*)-yl)-*N*-(4-(phenyldiazenyl)phenyl)benzamide; (E)-3: (*E*)-1,1’-(4-(*p*-tolyldiazenyl)-1,3-phenylene)bis(1*H*-pyrrole-2,5-dione); (Z)-3: (*Z*)-1,1’-(4-(*p*-tolyldiazenyl)-1,3-phenylene)bis(1*H*-pyrrole-2,5-dione); (E)-4: (*E*)-1,1’-(4-(*o*-tolyldiazenyl)-1,3-phenylene)bis(1*H*-pyrrole-2,5-dione); (Z)-4: (*Z*)-1,1’-(4-(*o*-tolyldiazenyl)-1,3-phenylene)bis(1*H*-pyrrole-2,5-dione). The synthesis and characterization details (experimental part) of these azobenzene derivatives containing maleimide moieties have been reported previously (Airinei et al. 2010; Rusu et al. 2011; Hulubei & Buiceac 2005; Airinei et al. 2011a,2011b).

### Computational details

All calculations based on semi-empirical molecular orbital theory have been carried out using HyperChem Release 8.0.6 molecular modeling software. The structures of the investigated azobenzene molecules in ground state were optimized using AM1 and PM3 semi-empirical methods with restricted Hartree-Fock (RHF) basis. All calculations referred to isolated molecule (gas phase). In this respect, the conjugate gradients algorithm (Polak-Ribier) was employed for the geometry optimization using a convergence set to the value of 0.01 kcal/(Å mol). The geometry optimization was done by minimization of the binding energy of the molecule. During energy minimization, it was searching for a molecular structure in which the energy did not change with infinitesimal changes in geometry and, respectively, the total root-mean-squared gradient (RMS-gradient) was close to zero. The optimal geometries of (E) and (Z) isomers were figured out starting from different initial conformers. The theoretical electronic absorption spectra of molecules were calculated on the optimized ground-state geometries using configuration-interaction (CI) method included in HyperChem.

In addition, Gaussian 03 program has been employed for more accurate calculation of the geometrical parameters of the molecules in ground state. To this end, the higher level quantum mechanics (QM) methods were employed, using the basis set 6-31+G(d,p) (or 6-31+G**) with both polarization and diffuse functions, i.e. ab-initio (RHF/6-31+G(d,p)) and density functional theory (B3LYP/6-31+G(d,p)). Note that, polarization functions significantly improve the description of molecular geometries (bond lengths and angles) as well as molecular relative energies (Ramachandran et al. 2008). In fact, polarization functions add flexibility within basis set, effectively allowing molecular orbitals to be more asymmetric about the nucleus. The adding of diffuse function, represented by the “+” sign, is useful to take into account the effect of the electrons when they are far from the nucleus.

All programs have been running on Windows 7 operating system using a computer with the following characteristics: Intel Core i7, 2.4 GHz, 16GB RAM.

## Results and discussion

### Optimized geometries and structural parameters

Table 1 summarizes molecular information about the molecules subjected to modeling as well as some parameters computed by HyperChem. According to the results from Table 1, azobenzene derivatives containing maleimide groups are molecules with appreciable values of polarizabilities (30–43 Å^3^) and refractivities (82–116 Å^3^) in their ground electronic state. The positive values of calculated partition coefficient (log *P*) suggest that azobenzene derivatives of concern are of hydrophobic nature.

**Table 1:** **Some molecular information about modeled azo-derivatives**

Compound	1	2	3	4
Empirical formula	C_16_H_11_N_3_O_2_	C_23_H_16_N_4_O_3_	C_21_H_14_N_4_O_4_	C_21_H_14_N_4_O_4_
Molecular mass, (a.m.u.)	277.28	396.40	386.37	386.37
Number of electrons (valence electrons + lone pairs)	102	146	142	142
Number of double occupied molecular orbitals (OMO)	51	73	71	71
Number of unoccupied (virtual) molecular orbitals (UMO)	44	63	59	59
Number of total molecular orbitals (OMO+UMO)	95	136	130	130
Polarizability, (Å^3^)	30.05	42.98	39.78	39.78
Refractivity, (Å^3^)	82.34	115.60	109.35	109.35
Log *P*	3.57	4.34	3.27	3.27

**Figure 2:**
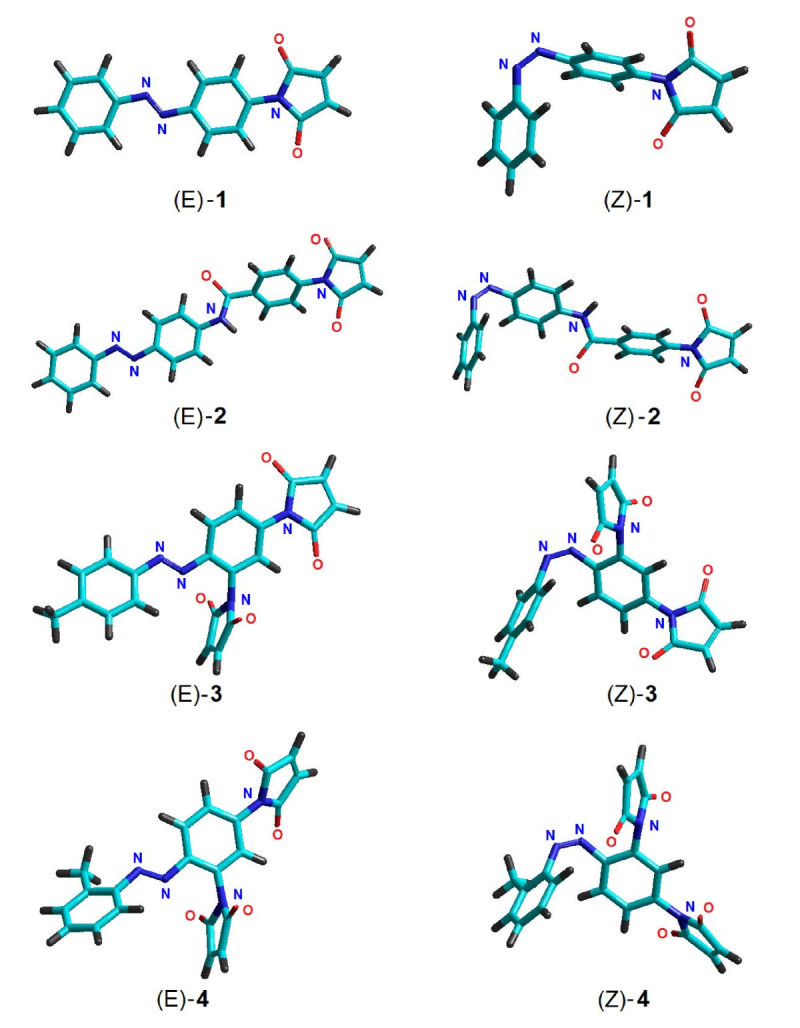
**Optimized structures of azobenzene derivatives computed by PM3 method: tubes rendering model.**

Figure 2 shows 3D-optimized molecular structures of the azobenzene derivatives, (E) and (Z) isomers, in ground state computed with PM3 method. Likewise, the optimal geometries has been computed and by AM1 method. Table 2 compiles information on binding energies, gradients and heats of formation calculated by semi-empirical methods for each optimized conformer. The results of computation have revealed that (E)-1 is planar according to both AM1 and PM3, while (E)-2 is planar only according to AM1 model. These planar conformations correspond to C_S_ molecular point group. All other investigated conformations are non-planar and correspond to C_1_ molecular point group.

**Table 2:** **Calculated energy, gradient and heat of formation by semi-empirical methods for 1-4**

Compound	(E) isomer		(Z) isomer	
	AM1	PM3	AM1	PM3
1				
Binding Energy, (kcal/mol)	-3681.5595	-3715.5188	-3686.5290	-3715.2679
RMS Gradient, (kcal/(Å mol))	0.0004869	0.0007137	0.005422	0.008860
Heat of formation (kcal/mol)	83.9204	49.9612	78.9509	50.2120
Molecular point group	C_S_	C_S_	C_1_	C_1_
2				
Binding Energy, (kcal/mol)	-5313.0421	-5355.8834	-5319.0632	-5354.1534
RMS Gradient, (kcal/(Å mol))	0.0001445	0.0010867	0.007611	0.009925
Heat of formation (kcal/mol)	81.7368	38.8955	75.7157	40.6255
Molecular point group	C_S_	C_1_	C_1_	C_1_
3				
Binding Energy, (kcal/mol)	-4945.5461	-5009.5677	-4951.5251	-5009.1396
RMS Gradient, (kcal/(Å mol))	0.0007359	0.0013178	0.009186	0.009233
Heat of formation (kcal/mol)	62.8079	-1.2137	56.8288	-0.7856
Molecular point group	C_1_	C_1_	C_1_	C_1_
4				
Binding Energy, (kcal/mol)	-4944.8592	-5009.6008	-4951.6597	-5008.1308
RMS Gradient, (kcal/(Å mol))	0.0004231	0.0007584	0.008362	0.009835
Heat of formation (kcal/mol)	63.4948	-1.2468	56.69429	0.223173
Molecular point group	C_1_	C_1_	C_1_	C_1_

To determine which form is more stable (E) or (Z), the relative strain energy (*E*_*rel*_) was calculated for each compound as the difference between binding energies of the isomers (E) and (Z). The relative strain energy (*E*_*rel*_) may be also calculated and as the difference between heats of the formation of isomers (Shaabani & Zahedi 2000). These computational results are focused in Table 3, pointing out that the energy is a function of molecular geometry. Thus, AM1 Hamiltonian shows that (Z) isomers are more stable, while PM3 model suggests that (E) isomers are the most stable. According to AM1 method, (Z) isomers are 4.97-6.80 kcal/mol more stable than (E) isomers. In contrast, PM3 model revealed that (E) isomers are 0.25-1.73 kcal/mol more stable than (Z) isomers. In order to disclose this inconsistency, we have done additional calculation of the relative strain energy (*E*_*rel*_) by MNDO semi-empirical method as well as by ab-initio RHF/6-31+G(d,p) and DFT/B3LYP/6-31+G(d,p). These results are also summarized in Table 3. Thus, the additional calculation using MNDO, ab-initio (RHF/6-31+G(d,p)) and DFT (B3LYP/6-31+G(d,p)) methods are in agreement with PM3 model, revealing the stability of (E) isomers. Hence, PM3 method has proved better performance compared to AM1 method for molecular geometry optimization of azobenzene derivatives (1–4), due to its improved description of steric effects. This may be attributed to the fact that PM3 model was developed using a largely mathematical optimization procedures for parameterization of two electron repulsion integrals. Thus PM3 model, uses a Hamiltonian that is very similar to the AM1 Hamiltonian, but the parameterization strategy is different. Whereas AM1 model was parameterized largely based on a small number of atomic data, PM3 was parameterized to reproduce a large number of molecular properties (Ramachandran et al. 2008).

**Table 3:** **Relative strain energy calculated for (E) isomer to (Z) isomer of azobenzene derivatives 1-4 by QM methods**

Compound	***E***_***rel***_, (kcal / mol)^a)^				
	AM1	PM3	MNDO	RHF / 6-31+G (d,p)	B3LYP / 6-31+G (d,p)
1	4.9695	-0.2508	-18.5492	-14.6837	-21.6789
2	6.0211	-1.7300	-1.9715	-16.8173	-22.3017
3	5.9791	-0.4281	-3.0078	-12.4247	-12.9393
4	6.8006	-1.4700	-3.1976	-14.3699	-12.5628

a) E_rel_ = E_E_ - E_Z_ = H_E_ - H_Z_.

Table 4 summarizes the selected structural parameters calculated by semi-empirical methods for all molecules studied (1–4) in their isomeric forms (E) / (Z). The selected geometrical parameters reported in Table 4 involve bond lengths, valence angles and torsion angles related to azo group (-N=N-) and adjacent carbon atoms (Csp^2^). It can be observed from Table 4 that the calculated bond length of azo group N=N is larger for (E) isomers than for (Z) isomers. For instance, according to AM1 computations the bond length of N=N is of 1.2313 Å for (E)-1 isomer and of 1.2044 Å for (Z)-1 isomer. In contrast, the bond lengths C-N and N-C are larger for (Z) isomers than for (E) isomers. Likewise, the semi-empirical calculations revealed that the interatomic angles C-N=N and N=N-C are higher for (Z) isomers comparing with (E) isomers. For example, the valence angle C-N=N for (Z)-1 is of 129.46° and 127.08° according AM1 and PM3, respectively, while for (E)-1 isomer the same angle C-N=N is much smaller being of 119.73° (AM1) and 119.88° (PM3). The dihedral angle (torsion angle) C-N=N-C is close to 180° or -180° for all (E) isomers, while for (Z) isomers this torsion angle is in the range of about ± 3°.

**Table 4:** **Summary of selected structural parameters related to azo-group atoms, for the compounds 1-4**

Compound	Method	Bond lengths			Angles		Torsions		
		C-N	N=N	N-C	C-N=N	N=N-C	C-C-N=N	C-N=N-C	N=N-C-C
(E)-1	AM1	1.4361	1.2313	1.4343	119.72	119.65	180	180	180
	PM3	1.4468	1.2319	1.4455	119.88	119.86	180	180	180
(Z)-1	AM1	1.4417	1.2044	1.4409	129.46	129.39	-139.327	2.241	45.313
	PM3	1.4520	1.2160	1.4524	127.08	127.05	-121.345	0.218	77.109
(E)-2	AM1	1.4363	1.2315	1.4332	119.68	119.73	180	180	180
	PM3	1.4471	1.2319	1.4452	119.78	119.90	169.061	179.896	174.997
(Z)-2	AM1	1.4434	1.2042	1.4386	129.37	129.58	-117.574	1.683	31.229
	PM3	1.4527	1.2161	1.4517	127.06	127.19	-95.658	0.187	61.950
(E)-3	AM1	1.4362	1.2302	1.4364	119.47	119.36	155.701	178.181	-26.792
	PM3	1.4458	1.2313	1.4466	119.71	119.75	-152.291	-179.476	11.429
(Z)-3	AM1	1.4427	1.2038	1.4427	128.80	128.99	122.991	-2.975	-50.910
	PM3	1.4511	1.2168	1.4522	127.43	127.53	-111.732	0.896	48.897
(E)-4	AM1	1.4390	1.2301	1.4344	118.50	120.22	41.406	-177.189	-1.464
	PM3	1.4476	1.2303	1.4482	119.48	119.75	45.337	-179.005	15.011
(Z)-4	AM1	1.4442	1.2037	1.4429	128.73	128.83	71.141	3.149	50.208
	PM3	1.4517	1.2168	1.4523	127.60	127.57	75.351	1.815	49.968

**Figure 3:**
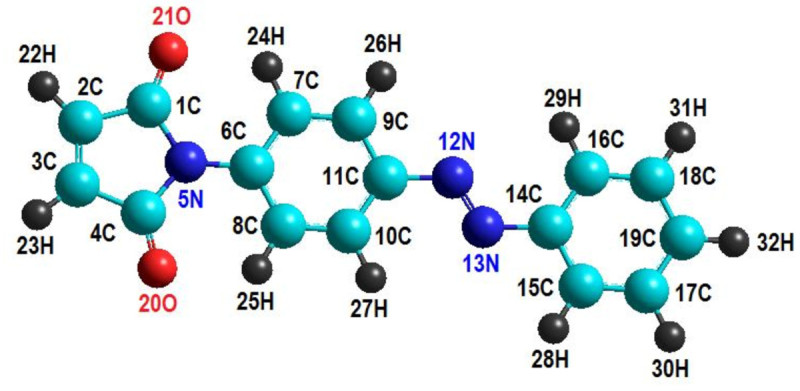
**Optimized molecular structures of (E)-1 azobenzene derivative, in ground state (S**_**0**_ **), computed by AM1 method: - balls and sticks rendering model with full atomic numbering.**

Compound (E)-1 was obtained experimentally in pure state as a light orange crystalline solid and its crystallographic data and experimental geometric parameters were reported (Rusu et al. 2011). It has been mentioned that the compound 1 (C_16_H_11_N_3_O_2_) displays a (E) conformation with respect to azo group and the molecule is non-planar. Thus, one of the goals of this work was to calculate geometrical parameters for (E)-1 and to compare with available experimental data. In this respect, in addition to HyperChem semi-empirical calculations (AM1 and PM3), the Gaussian program has been employed for more accurate computations of the geometrical parameters in ground state using higher level QM methods, such as ab-initio (RHF/6-31+G(d,p)) and density functional calculations (DFT/B3LYP/6-31+G(d,p)). Figure 3 shows the optimized molecular structure of (E)-1 azobenzene derivative (C_16_H_11_N_3_O_2_) computed by AM1 model. In this figure, the ball and stick rendering model with full atomic numbering has been used for spatial representation.

**Figure 4:**
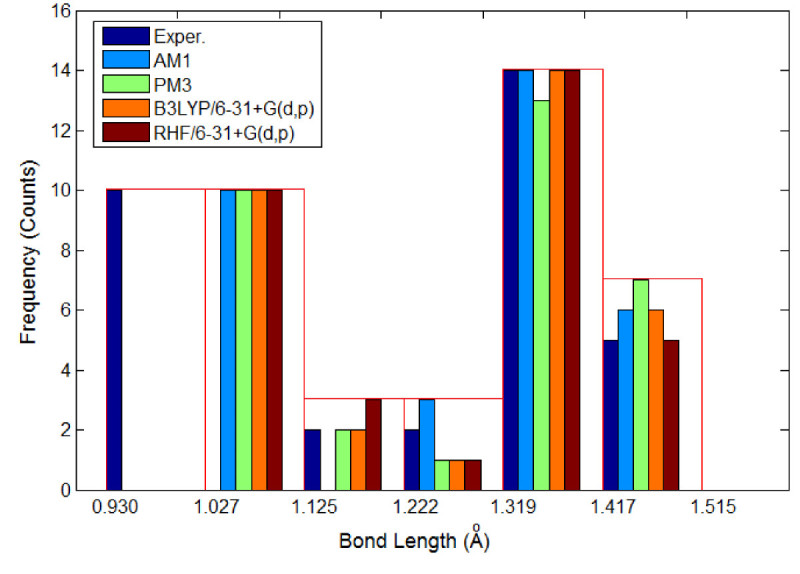
**Histogram of bonds length distribution determined for (E)-1 azobenzene derivative: crystallographic data (experimental) versus QM-calculations.**

**Figure 5:**
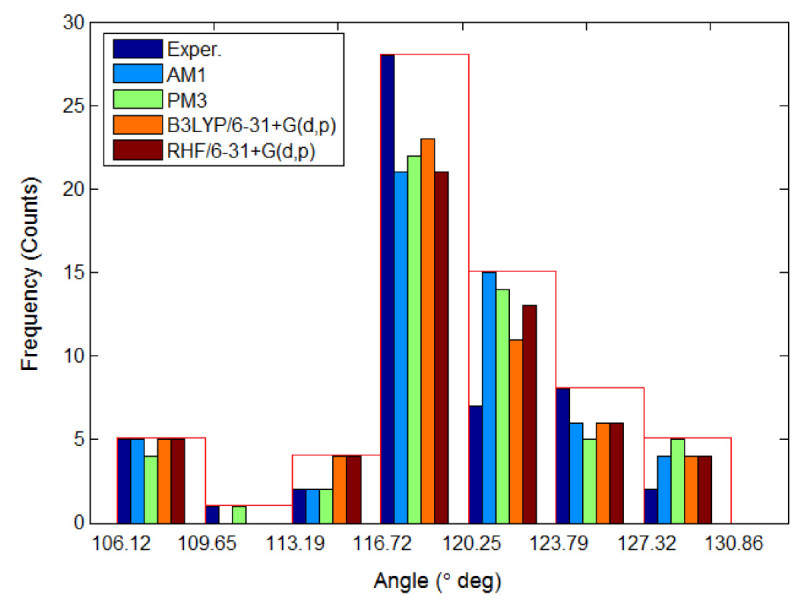
**Histogram of interatomic angles distribution determined for (E)-1 azobenzene derivative: crystallographic data (experimental) versus QM-calculations.**

Table 5 summarizes the selected bond lengths and interatomic angles for (E)-1 (C_16_H_11_N_3_O_2_) computed by quantum-mechanics methods (QM) and compared with experimental values. Note that in Table 5 only a part of geometrical parameters are reported. In fact, a total number of 33 bond lengths and 53 interatomic angles have been determined by X-ray structure analysis (Rusu et al. 2011), and the corresponding counterparts of these geometrical parameters have been calculated in this work by QM methods. For a more compact representation, all these results (experimental and calculated) are shown as histograms in Figures 4 and 5. These histograms illustrate the distributions of bond lengths and valence angles for (E)-1 molecule in accordance with the experimental observations and theoretical models. Both experimental evidences and theoretical computations revealed that the shortest bond lengths were attributed to the bonds C-H. According to X-ray structure analysis C-H bond is of length of 0.93Å whereas the theoretical models predict a larger bond length for C-H of about 1.07 – 1.09 Å. The largest bond length in the molecule (E)-1 is attributed to C-C bond from maleimide group. Thus, according to experimental evidence the largest bond length is of 1.4893 Å being ascribed to 4C-3C bond. The theoretical models also predict that 4C-3C bond has the largest length being of 1.5138 Å, 1.4937 Å and 1.4952 Å according to AM1, RHF/6-31+G(d,p) and B3LYP/6-31+G(d,p) methods, respectively. In accordance with semi-empirical method PM3, the largest bond length is 1C-2C with the value of 1.5039 Å. The histogram from Figure 4 shows that the highest frequency corresponds to the bin interval of 1.319-1.417 Å, being attributed to C-C and C-N bond types. Regarding interatomic angles, the smallest valence angle given by X-ray structure analysis is of 106.12° and corresponds to 5N-4C-3C from maleimide group. According to theoretical geometry optimization calculations using AM1, RHF/6-31+G(d,p) and B3LYP/6-31+G(d,p) methods, the valence angle 5N-4C-3C is also the smallest one and has the following values of 107.12°, 107.17° and 107.21°, respectively. The PM3 computations suggest that the smallest valence angle in molecule (E)-1 is 5N-1C-2C from maleimide group, being of 106.51°. In accordance with X-ray-structure analysis of (E)-1, the largest interatomic angle is 21O-1C-2C, which has the value of 128.17°. The theoretical models predict for this angle 21O-1C-2C the values ranging from 125.04° to 127.51°. According to optimized geometries computed by QM methods the largest valence angle is 2C-3C-23H which is equal to theoretical values of 130.85° (AM1), 128.37° (PM3), 130.20° (RHF/6-31+G(d,p)) and 130.15° (B3LYP/6-31+G(d,p)). The histogram shown in Figure 5 indicates that the highest frequency (count) corresponds to the bin interval of 116.72°-120.25°, which is mainly attributed to the interatomic angles of type C-C-H and C-C-C.

**Table 5:** **Selected bond lengths (in Å) and interatomic angles (in deg°) computed for (E)-1 (** C_16_H_11_N_3_O_2_**) by QM methods and comparison with experimental values**

Geometry	Experim^a)^	AM1	PM3	RHF 6-31+G (d,p)	B3LYP 6-31+G (d,p)
*Bond*					
12N-13N	1.2539	1.2313	1.2319	1.2183	1.2589
12N-11C	1.4320	1.4343	1.4455	1.4192	1.4161
5N-1C	1.4046	1.4329	1.4581	1.4021	1.4219
5N-4C	1.4049	1.4330	1.4583	1.4023	1.4223
5N-6C	1.4346	1.4108	1.4420	1.4356	1.4351
20O-4C	1.2062	1.2302	1.2127	1.1864	1.2137
7C-9C	1.3845	1.3876	1.3889	1.3864	1.3926
7C-6C	1.3918	1.4143	1.4003	1.3916	1.4062
7C-24H	0.9300	1.1024	1.0972	1.0666	1.0787
13N-14C	1.4252	1.4360	1.4468	1.4216	1.4189
10C-8C	1.3781	1.3889	1.3862	1.3805	1.3885
10C-11C	1.396	1.4070	1.3994	1.3897	1.4042
10C-27H	0.9300	1.1028	1.0972	1.0725	1.0837
8C-6C	1.3893	1.4134	1.4033	1.3993	1.4117
9C-11C	1.3874	1.4104	1.3997	1.3821	1.3992
9C-26H	0.9300	1.1028	1.0973	1.0747	1.0855
14C-15C	1.3879	1.4129	1.4012	1.3863	1.4025
14C-16C	1.3952	1.4093	1.3992	1.3937	1.4072
21O-1C	1.2044	1.2302	1.2099	1.1865	1.2138
*Angle*					
13N-12N-11C	112.96	119.65	119.86	115.83	115.13
1C-5N-4C	109.32	108.15	107.37	108.25	107.86
1C-5N-6C	125.35	125.93	128.18	125.78	125.99
4C-5N-6C	125.18	125.91	124.44	125.96	126.14
9C-7C-6C	119.44	121.03	119.88	119.90	119.82
9C-7C-24H	120.30	117.68	118.25	119.06	119.65
6C-7C-24H	120.30	121.28	121.87	121.03	120.53
12N-13N-14C	113.94	119.72	119.88	115.95	115.27
8C-10C-11C	120.04	121.19	119.81	120.56	120.64
8C-10C-27H	120.00	118.26	119.01	119.46	119.99
11C-10C-27H	120.00	120.54	121.18	119.98	119.36
10C-8C-6C	119.80	120.92	120.44	120.52	120.41
10C-8C-25H	120.10	117.76	120.43	118.82	119.43

a) Experimental X-ray crystal data from (Rusu et al. 2011).

In order to estimate the goodness-of-fit between experimental and theoretical parameters (i.e. bond lengths, valence angles) corresponding to (E)-1 molecule, the residual error functions (deviation errors) have been computed in terms of root-mean-square deviation (*RMSD*) and average relative error (*ARE*) as follows:

1 $$\;\text{RMSD}=\sqrt{\frac{1}{\left(N-1\right)}\sum_{i-1}^{N}{\left(X_{i}^{\text{calc}}-X_{i}^{\exp}\right)}^{2}}$$

2 $$\text{ARE}=\frac{100}{N}\sum_{i=1}^{N}\left(\frac{\left|X_{i}^{\exp}-X_{i}^{\text{calc}}\right|}{X_{i}^{\exp}}\right)$$

where *N* is the number of data, *X*^*exp*^ denotes the experimental parameter (i.e. bond length, valence angle, etc.) and *X*^*calc*^ is the theoretical parameter calculated by QM method. Table 6 summarizes the values of *RMSD* and *ARE* to compare the abilities of QM methods for prediction and fitting experimental data related to bond lengths and angles of (E)-1 azobenzene derivative. According to the results reported in Table 6, the best methods for predicting the structural parameters of (E)-1 are ab-initio at the level RHF/6-31+G(d,p) and DFT/B3LYP/6-31+G(d,p) followed by semi-empirical methods PM3 and AM1. Thus, ab initio calculations atRHF/6-31+G(d,p) level bestowed more accurate results on (E)-1 structure giving a deviation error from crystallographic data of about 5.00% for bond lengths and 0.97% for interatomic angles. The density functional computations at the level B3LYP/6-31+G(d,p) have predicted the geometry of (E)-1 in gas phase with the deviation errors from crystallographic data of about 5.60% for bond lengths and 0.95% for valence angles. In case of semi-empirical calculations of structural parameters by AM1 and PM3 methods, the residual errors are of 6.44%, 6.11% for bond lengths and 1.33%, 0.94% for interatomic angles, respectively (Table 6). Thus, PM3 method predicts better the structural parameters of (E)-1 comparing with AM1. All calculations (AM1, PM3, RHF/6-31+G(d,p) and B3LYP/6-31+G(d,p)) performed in this work have revealed that the molecule (E)-1 is planar from theoretical standpoint. In contrast, the X-ray data offered a non-planar geometry for the same molecule (Rusu et al. 2011). For instance, the torsion angle 1C-5N-6C-8C is of 180° according to the theoretical predictions, but the same dihedral angle is of 140.32° according to crystallographic data. Also, it has been reported by X-ray structure analysis a torsion angle of 21.5° for 13N-12N-11C-10C (Rusu et al. 2011), whereas our theoretical computations have indicated for the same dihedral angle a value of 0°. All four planar geometries of (E)-1 computed by QM methods have converged to the same values of torsion angles. The discrepancy between theory and experiment on the planar geometry of (E)-1 may be attributed to the fact that all theoretical computations have been performed for an isolated molecule in vacuum. By contrast, according to experimental observations (i.e. crystallographic data) the molecules of (E)-1 in the crystal are stacked along the [100] direction with a mean interplanar distance of 3.857(1) Å and, additionally, C-H…O interactions link them into double layers parallel to the ac-plane (Rusu et al. 2011). Such interactions existing into the crystal may affect the planarity of (E)-1, turning the geometry of this molecule into the non-planar one.

**Table 6:** **Root mean square deviation (*RMSD*) and average relative error (*ARE*) used for ascertaining the goodness-of-fit between observed and predicted bond lengths and angles for the molecule (E)-1 (** C_16_H_11_N_3_O_2_**)**

	AM1	PM3	B3LYP / 6-31+G(d,p)	RHF / 6-31+G(d,p)
*Bond lengths*				
*RMSD*, (Å)	0.3567	0.3428	0.3111	0.2401
*ARE*, (%)	6.4427	6.1093	5.5991	5.0003
*Interatomic angles*				
*RMSD*, (deg °)	1.7406	1.7903	0.4967	0.6867
*ARE*, (%)	1.3338	0.9386	0.9478	0.9692

### Charge distribution, electrostatic potential and QSAR properties

**Figure 6:**
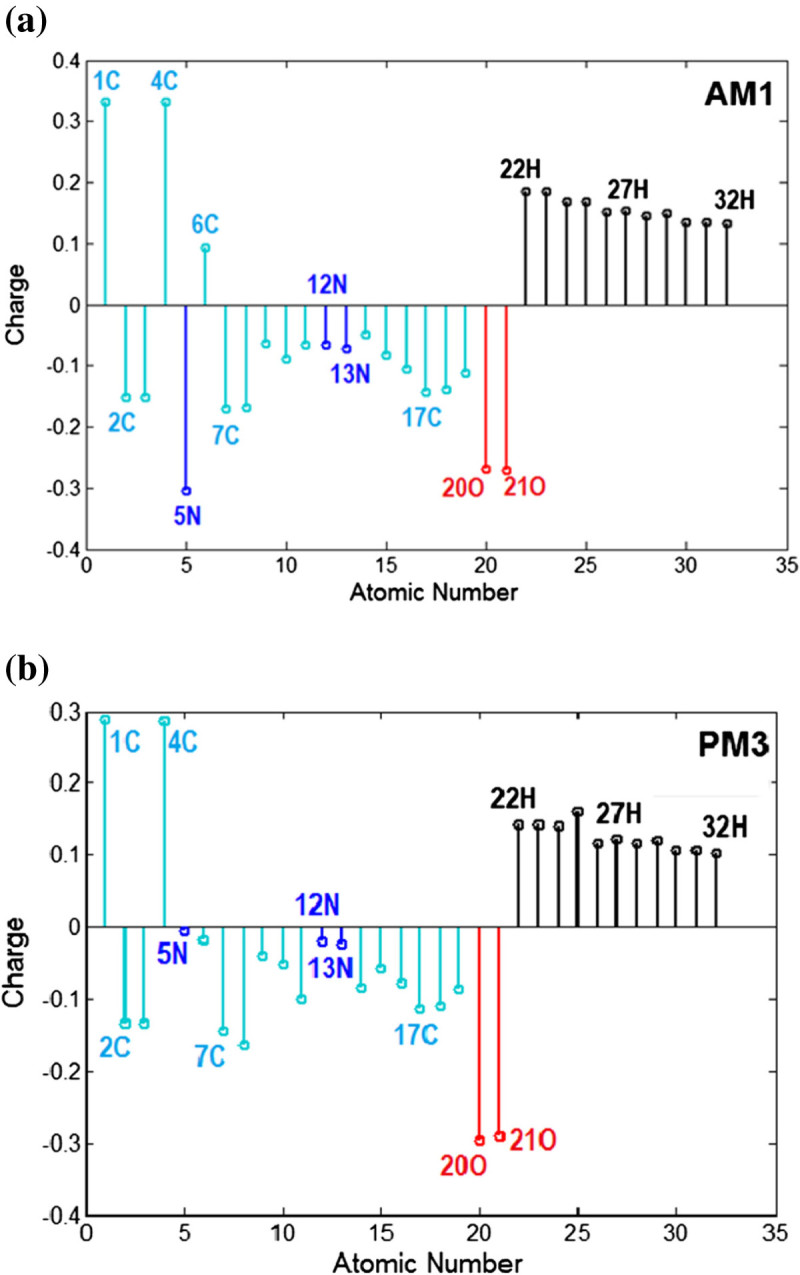
**Atomic charges distribution in molecule (E)-1 computed by semi-empirical methods: (a) AM1 and (b) PM3.**

The population analysis in computational chemistry stands for estimating by calculation of the partial atomic charges of the studied molecule (Ramachandran et al. 2008). The Mulliken population analysis is the most common type of such computation and HyperChem makes use of it. The atomic charge distribution in the molecule is of great importance and has a significant impact on electrostatic potential, dipole moment and theoretical absorption spectra. Figure 6 shows the atomic charges distribution in molecule (E)-1 computed by semi-empirical methods AM1 (Figure 6a) and PM3 (Figure 6b). As one can see from Figure 6, the highest positive net charges are attributed to 1C and 4C atoms. All hydrogen atoms have positive charges. The oxygen heteroatoms 20O and 21O are assigned with strong negative charges. Also, nitrogen heteroatoms 5N, 12N and 13N have the negative charges that are stronger according to AM1 computations comparing with PM3. Note that, the atomic charges distributions have been calculated for all azobenzene derivatives (1–4). In this respect, Table 7 gives the net charges for selected central atoms C-N=N-C related to azo group and the adjacent carbons of sp^2^ hybridization (Csp^2^). According to both semi-empirical methods (AM1 and PM3), the calculated atomic charges for azo nitrogen atoms (N=N) are smaller in case of all (E) isomers as compared to (Z) isomers. The switching to (Z) isomers will increase the net charges of nitrogen atoms from azo group (N=N). For example, according to AM1 calculations for (E)-1 molecule, the net charges of nitrogen atoms (N=N) are equal to -0.0662 and -0.0723. The switching to (Z)-1 isomer will increase the atomic charges of nitrogen atoms to the values +0.0061 and +0.0031, respectively. The carbon atoms C-sp^2^ linked to azo group are more negatively charged for (Z) isomers comparing with (E) isomers (Table 7).

**Table 7:** **Net charges of atoms related to azo group and adjacent carbons (C-N=N-C) computed by semi-empirical methods for 1–4**

Compound		C (sp^2^)	N	N	C (sp^2^)
(E)-1	AM1	-0.0653	-0.0662	-0.0723	-0.0482
	PM3	-0.1006	-0.0192	-0.0249	-0.0834
(Z)-1	AM1	-0.1206	0.0061	0.0031	-0.1091
	PM3	-0.1063	0.0334	0.0386	-0.1177
(E)-2	AM1	-0.0806	-0.0654	-0.0757	-0.0458
	PM3	-0.1082	-0.0180	-0.0263	-0.0810
(Z)-2	AM1	-0.1441	0.0102	-0.0040	-0.0996
	PM3	-0.1274	0.0347	0.0350	-0.1090
(E)-3	AM1	-0.0523	-0.0675	-0.0524	-0.0519
	PM3	-0.0859	-0.0351	-0.0008	-0.0905
(Z)-3	AM1	-0.1122	0.0006	0.0187	-0.1148
	PM3	-0.1062	0.0077	0.0542	-0.1297
(E)-4	AM1	-0.0609	-0.0635	-0.0583	-0.0465
	PM3	-0.0745	-0.0347	0.0083	-0.0812
(Z)-4	AM1	-0.1120	0.0035	0.0197	-0.1090
	PM3	-0.1059	0.0074	0.0574	-0.1224

**Figure 7:**
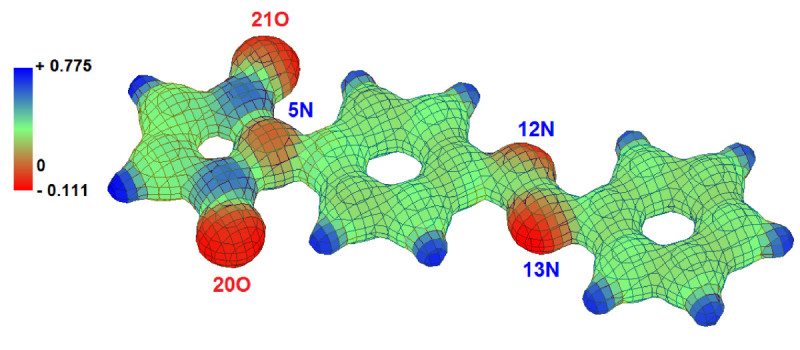
**Isosurface plot of the electrostatic potential (ESP) in the spatial vecinity of (E)-1 azobenzene derivative: AM1 computation results.**

Based on atomic charge distributions, the *electrostatic potential surface* (ESP) surrounding the molecule has been computed by HyperChem using a grid-points technique. Electrostatic potential is useful for finding sites of reaction in a molecule: positively charged species (e.g. H^+^) tend to attack the sites where the electrostatic potential is strongly negative (electrophilic attack). Figure 7 illustrates a three-dimensional (3D) mapped isosurface of the electrostatic potential surrounding (E)-1 molecule that has been computed at the level of AM1 method. The red colors indicate negative ESP regions followed by green colors which denote slightly positive ESP regions and blue colors indicating strong positive ESP regions. As one can see from Figure 7, oxygen heteroatoms 20O and 21O have the highest negative ESP regions, being the sites for electrophilic attack. Also, the isosurface plot shows that nitrogen heteroatoms 12N and 13N of azo bridge have more negative ESP regions than nitrogen heteroatom 5N from maleimide group. Whenever lone pair (*n*)-containing heteroatoms are part of a double-bonded system, they may undergo protonation under acidic conditions. Thus, the computed ESP predicts that in the acidic medium, the protonation reaction will occur preferable on nitrogen atoms from azo group and not on nitrogen atom from maleimide moiety. The electrostatic potential levels (i.e. minimum and maximum values) for all azobenzene derivatives (1–4) are reported in Table 8. In addition, Table 8 lists the dipole moment values as well as some *quantitative structure-activity relationship* (QSAR) properties computed at the level of semi-empirical Hamiltonians (AM1 and PM3). The results presented in Table 8 reveal that, dipole moments values (*DM*) are higher for (Z) isomers than for (E) isomers. For instance, in case of (E)-1 isomer the dipole moment is of 2.052 D according to AM1 method, whereas for (Z)-1 isomer *DM* value is higher being equal to 4.755 D (AM1). The surface areas and volumes of molecules (1–4) have been calculated by numerical integration grid techniques included in HyperChem. The computational results from Table 8 indicate that surface areas and volumes are higher for (E) isomers than for corresponding (Z) isomers. According to X-ray-structure analysis reported in Ref. (Rusu et al. 2011) the molecular volume for the isomer (E)-1 is about 650.18 (Å^3^), which is smaller about 24% than the predicted volumes calculated at the level of semi-empirical Hamiltonians. The hydration energy (*E*_*H*_) calculated for azobenzene derivatives (1–4) ranged from -13.06 kcal/mol to -5.91 kcal/mol (Table 8).

**Table 8:** **Dipole moments, electrostatic potential levels and some QSAR properties of 1–4, computed at the level of semi-empirical methods**

Compound		***DM***(Debye)	ESP-min	ESP-max	a)Size, (Å)	Surf. area, (Å^2^)	Volume, (Å^3^)	E_H_, (kcal/mol)
(E)-1	AM1	2.052	-0.111	0.775	14.977	498.66	810.26	-8.78
	PM3	1.983	-0.115	0.491	15.023	498.21	808.06	-8.59
(Z)-1	AM1	4.755	-0.104	0.075	12.379	486.23	792.71	-9.61
	PM3	4.252	-0.094	0.069	12.012	498.21	807.64	-9.49
(E)-2	AM1	5.652	-0.120	0.122	21.538	666.20	1113.99	-12.24
	PM3	4.729	-0.140	0.521	21.415	675.13	1126.70	-12.10
(Z)-2	AM1	7.173	-0.124	0.318	17.963	658.69	1105.88	-13.06
	PM3	5.517	-0.130	0.498	16.894	674.06	1120.41	-12.99
(E)-3	AM1	1.091	-0.127	0.350	15.888	624.97	1056.08	-6.22
	PM3	0.723	-0.130	0.485	15.821	628.07	1054.64	-6.09
(Z)-3	AM1	3.598	-0.116	0.370	13.044	605.12	1033.44	-6.43
	PM3	3.142	-0.123	0.562	12.638	620.13	1045.44	-6.33
(E)-4	AM1	1.761	-0.118	0.105	14.954	612.69	1041.26	-5.92
	PM3	0.642	-0.124	0.500	14.893	627.96	1061.31	-5.91
(Z)-4	AM1	3.237	-0.120	0.309	11.855	599.65	1023.35	-6.28
	PM3	2.818	-0.124	0.496	11.488	607.57	1034.30	-6.17

a) Computed as the maximum atomic distance in the molecule.

### Electronic properties

**Figure 8:**
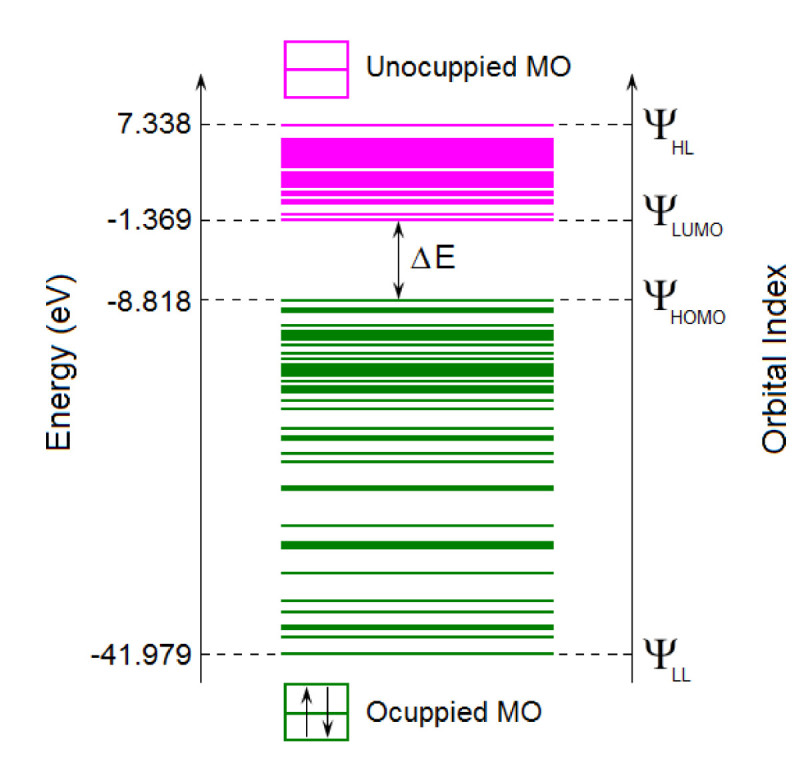
**Energy spectrum of molecular orbitals computed by AM1 method for (E)-1 azobenzene derivative, in its ground state (S**_**0**_ **).**

The information regarding number of electrons, number of double occupied molecular orbitals and virtual molecular orbitals are summarized in Table 1 for each studied azobenzene derivative (1–4). The energy spectra of molecular orbitals (MOs) in ground state have been computed for all molecules (1–4) by means of semi-empirical methods (AM1 and PM3). Figure 8 shows as an example the energy spectrum of MOs calculated for (E)-1 in its ground state (S_0_) at the level of AM1 model. As can be observed, for each molecular orbital the corresponding energy value (ϵ_j_-eigenvalue) and orbital index (Ψ_j_) are associated. The green lines indicate the eigenvalues of occupied molecular orbitals (OMO), while the pink lines denote the energy levels of unoccupied (virtual) orbitals (UMO). Such spectra give useful information about the energy values of frontier molecular orbitals and the corresponding energy gap *ΔE*.

The eigenvalues of *highest occupied molecular orbital* (HOMO) and *lowest unoccupied molecular orbital* (LUMO) give information about the chemical activity and stability of the molecule, and are important parameters from quantum chemistry standpoint. The frontier orbital HOMO as an electron donor reflects the ability of the molecule to donate an electron, whereas LUMO as an electron acceptor represents the ability to receive an electron (Jesintha John et al. 2012; Mahadevan et al. 2012).

Based on theoretical MOs energy spectra the following molecular descriptors have been computed, namely, ionization potential (*IP*), electron affinity (*EA*), electronegativity (*χ*), chemical hardness (*η*) and electrophilicity index (*ω*). Following the standard procedure based on Koopmans’ theorem, the ionization potential and electron affinity have been calculated from the energy values of the highest occupied and the lowest unoccupied molecular orbitals, respectively, i.e. *IP* ≈ - ϵ_HOMO_ and *EA* ≈ -ϵ_LUMO_ (Fayet et al. 2010; Ramachandran et al. 2008; Hurjui et al. 2013). The molecular descriptor related to electronegativity (*χ*) characterizes the electron donor/acceptor behavior of the molecule being calculated as (Fayet et al. 2010; Hurjui et al. 2013):

3 $$\chi =\frac{\text{IP}+\text{EA}}{2}$$

Note that electronegativity is equal, but opposite in sign, to the chemical potential (*μ*), i.e. *μ* = - χ. The chemical hardness (*η*) and electrophilicity index (*ω*) have been computed using the following formulas (Fayet et al. 2010; Hurjui et al. 2013):

4 $$\eta =\frac{\text{IP}-\text{EA}}{2}$$

5 $$\omega =\frac{\mu^{2}}{2\eta }$$

Table 9 presents the energy values of HOMO and LUMO orbitals, energy gap (*ΔE*) and molecular descriptors (*χ,η* and *ω*) calculated for azobenzene derivatives (1–4) at the level of semi-empirical Hamiltonians AM1 and PM3. The energetic values from Table 9 reveal that the energy gap (*ΔE=* ϵ_LUMO_*-* ϵ_HOMO_) of studied azobenzene derivatives (1–4) varies from 7.022 eV to 7.860 eV. In most of the cases, the calculated values of energy gap (*ΔE*), electronegativity (*χ*) and chemical hardness (*η*) are slightly higher for (Z) isomers than for (E) isomers.

**Table 9:** **Energies of frontier MOs, the energy gap and molecular descriptors computed at the level of AM1 and PM3 Hamiltonians for (1–4)**

Compound		ϵ_LUMO_, (eV)	ϵ_HOMO_, (eV)	***ΔE***, (eV)	***χ***, (eV)	***η***, (eV)	***ω***, (eV)
(E)-1	AM1	-1.369	-8.818	7.449	5.093	3.724	3.483
	PM3	-1.443	-8.927	7.484	5.185	3.742	3.592
(Z)-1	AM1	-1.397	-9.097	7.700	5.247	3.850	3.575
	PM3	-1.344	-9.138	7.794	5.241	3.897	3.524
(E)-2	AM1	-1.536	-8.558	7.022	5.047	3.511	3.627
	PM3	-1.457	-8.738	7.281	5.098	3.640	3.569
(Z)-2	AM1	-1.534	-8.834	7.300	5.184	3.650	3.681
	PM3	-1.457	-8.940	7.483	5.199	3.741	3.611
(E)-3	AM1	-1.324	-8.783	7.459	5.053	3.729	3.424
	PM3	-1.436	-9.001	7.565	5.218	3.782	3.600
(Z)-3	AM1	-1.366	-9.071	7.705	5.218	3.853	3.534
	PM3	-1.363	-9.134	7.771	5.248	3.886	3.545
(E)-4	AM1	-1.355	-8.884	7.529	5.120	3.765	3.481
	PM3	-1.332	-9.193	7.860	5.263	3.930	3.523
(Z)-4	AM1	-1.365	-9.136	7.771	5.250	3.885	3.548
	PM3	-1.374	-9.177	7.803	5.276	3.901	3.567

**Figure 9:**
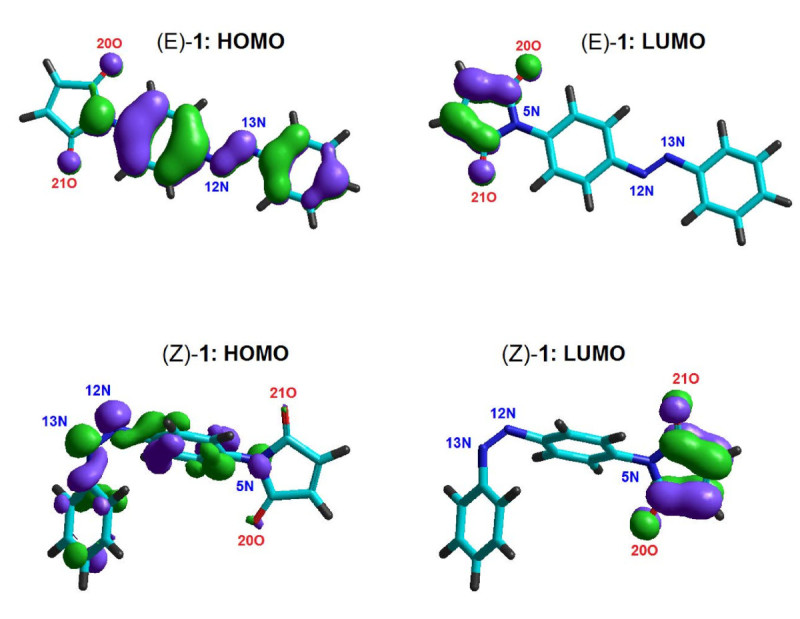
**Frontier orbital plots (HOMO-LUMO) for optimized geometries of azobenzene derivative isomers (E)-1 and (Z)-1 computed at the level of PM3 method; partial atomic numbering.**

The plots of HOMO and LUMO molecular orbitals for the isomers (E)-1 and (Z)-1 are shown as an example in Figure 9. The negative phase of wave-function is illustrated with blue color, while the positive phase with green color. As one can see, for both isomers (E)-1 and (Z)-1 the HOMO lobes are spread mainly over aromatic rings and azo group. In contrast, the LUMO lobes are almost uniformly distributed over maleimide moiety. Similar distribution of frontier orbitals have been obtained and for the other compounds 2–4.

### Simulated electronic absorption spectra

Electronic absorption spectra arise from transitions between electronic states of different quantum numbers induced by electromagnetic radiation with ultraviolet or visible (UV–vis) light (Grimme 2004). The organic molecules containing π-electrons and lone-pairs electrons (*n*-electrons) allow under UV–vis radiation (>200 nm) the electronic transitions of type n→π* and π→π*. The intensities of the bands corresponding to n→π* transitions are usually lower than those attributed for π→π* transitions (Kazitzyna & Kupletskaya 1971). In order to reveal electronic transitions, their energies and oscillator strengths, the theoretical absorption spectra play an important role (Mahadevan et al. 2012).

The singly-excited configuration interaction method (CI) at the level of semi-empirical Hamiltonians (AM1 and PM3) has been employed to calculate the theoretical absorption electronic spectra of the investigated azobenzene derivatives (1–4). Note that, configuration interaction (CI) involves the excitation of a single electron from one of the occupied orbital to one of the unoccupied orbital. For calculation of electronic spectra based on CI-method the HyperChem program has been used. In this respect, the energy criterion has been selected by setting the maximum excitation energy at the level of 10 eV, which is larger than the HOMO-LUMO gap of studied molecules (1–4). The computation of theoretical electronic spectrum by HyperChem involves the performing of CI calculation in conjunction with single point energy calculation. To this end, a self-consistent-field calculation (SCF) is first performed to obtain the reference electronic configuration associated with the singlet ground state of the molecule. Afterwards, HyperChem generates a series of singly excited configurations, computes the Hamiltonian matrix elements between them, and then diagonalizes the matrix to get the spectrum of electronic states (HyperChem 2002). Note that, the solvent effect has not considered for simulation of electronic spectra.

**Figure 10:**
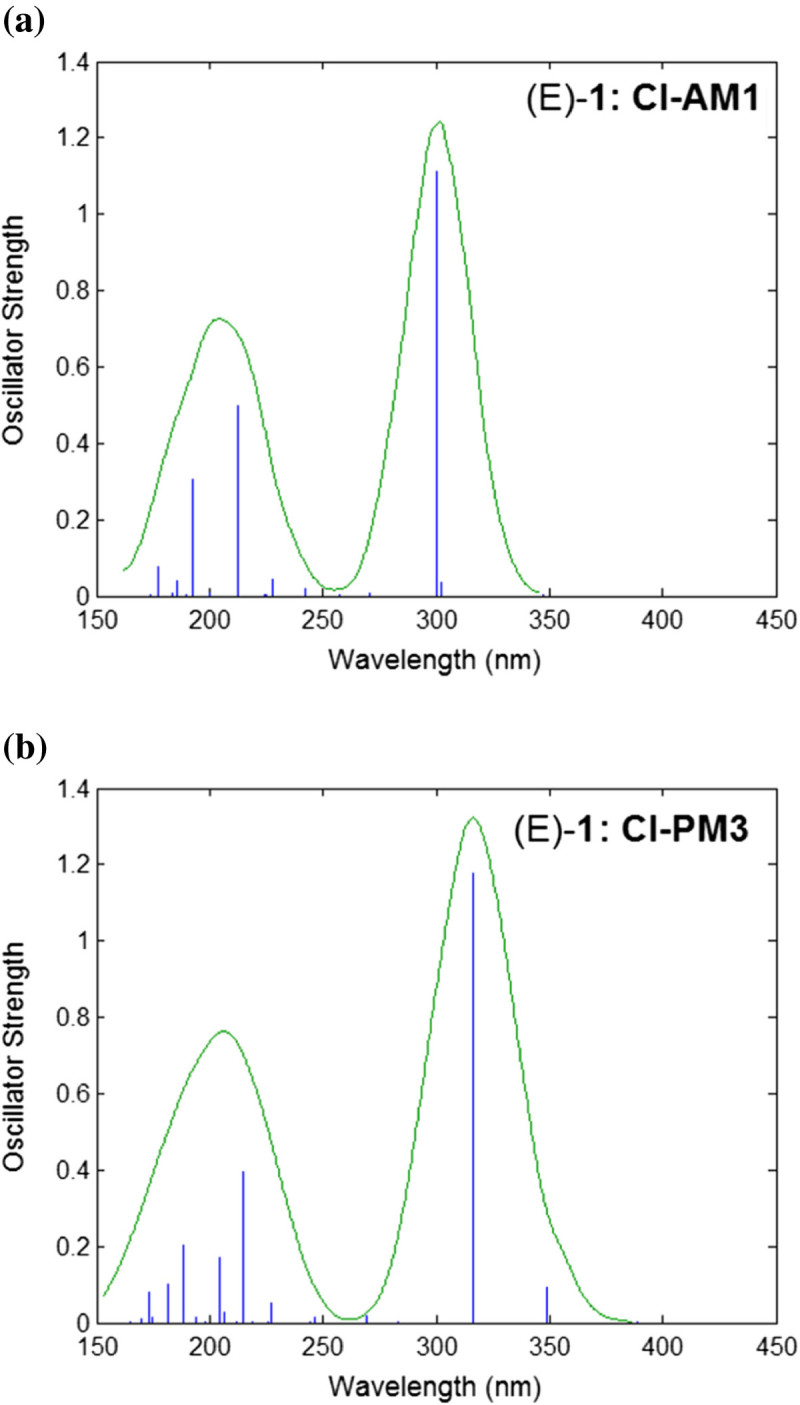
**Theoretical UV–vis absorption spectra for (E)-1 azobenzene derivative: CI-AM1 (a) and CI-PM3 (b) computation results.**

Figure 10 shows the theoretical UV–vis absorption spectra for (E)-1 isomer computed from a single point energy calculation at the level of semi-empirical Hamiltonians (AM1 and PM3) using singly-excited configuration interaction (CI). The simulated electronic absorption spectra are presented by plotting the transition energy given as *wavelength* versus their intensity given as *oscillator strength* (*f*). Transitions in the spectra are represented as separate vertical blue lines (peaks) denoting the excited electronic states that are spectroscopically active. These transitions are mainly of type *singlet (ground state)-to-singlet (excited state)*. Likewise, the plotted electronic spectra display the fitting line (green), which is formed by nonlinear fit, i.e. applying a Gaussian or Lorentzian distribution to each peak using a line width adjustable parameter (HyperChem 2002). As one can see from Figure 10a, the method CI-AM1 predicts for isomer (E)-1 the highest peak at 300.07 nm with oscillator strength (intensity) of *f*=1.113. This peak is assigned to the transition HOMO → LUMO_+1_. For the same isomer (E)-1, Figure 10b displays the electronic spectrum computed by CI-PM3 method. In this case, the highest peak appears at 316.11 nm with oscillator strength of *f*=1.175, being also assigned to the transition HOMO → LUMO_+1_.

**Figure 11:**
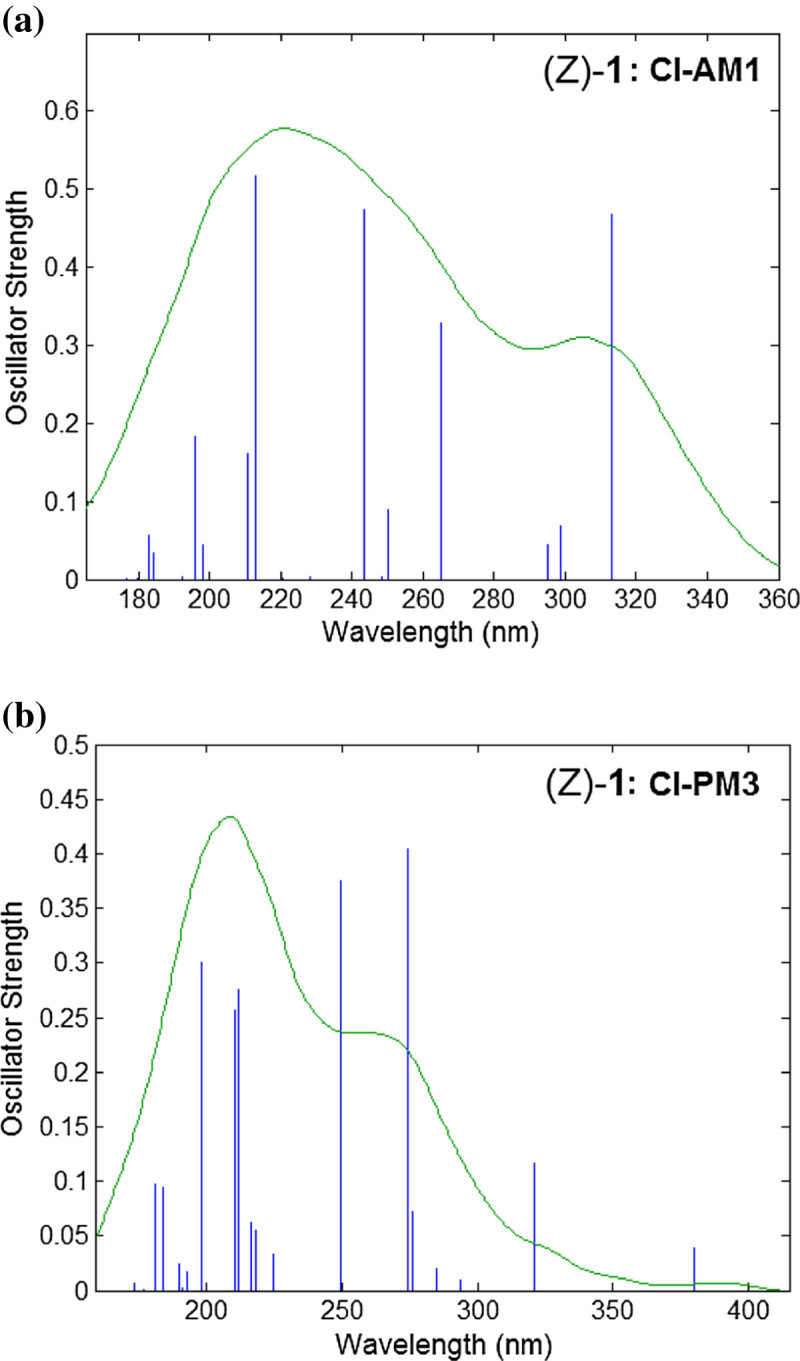
**Theoretical UV–vis absorption spectra for (Z)-1 azobenzene derivative: CI-AM1 (a) and CI-PM3 (b) computation results.**

Figure 11 shows the simulated electronic spectra for the isomer (Z)-1. By comparing the calculated electronic spectra of isomers (E)-1 (Figure 10) and (Z)-1 (Figure 11), one can see that the oscillator strengths are smaller in case of (Z)-1. The method CI-AM1 predicts for (Z)-1 a transition at 312.90 nm with oscillator strength of 0.467 (Figure 11a), being assigned to HOMO → LUMO_+1_. Figure 11b illustrates that according to CI-PM3 method, the highest peak appears at 274.51 nm with an intensity of *f*=0.405 attributed to the transition HOMO → LUMO_+1_.

For the compounds (1–4), the calculated wavelengths higher than 300 nm are of interest, since the experimental studies revealed the absorption bands of the π→π* or n→π* transitions in this domain. In this respect, Table 10 presents the theoretical wavelengths (*λ*) with values higher than 300 nm, their oscillator strengths (*f*) and excitation energies (*E*_*λ*_) as well as the corresponding spectral assignments calculated for (E) / (Z) isomers of compounds (1–4). As can be observed from the data reported in Table 10, for this spectral domain (*λ* > 300 nm) the oscillator strengths are higher for (E) isomers comparing with (Z) ones. Table 11 summarizes the maximum absorption wavelengths (*λ*_*max*_) determined experimentally from UV–vis absorption spectra of studied compounds (1–4) in various solvents. Thus, according to the data presented in Table 11, the absorption bands have been observed for (1–4) in the spectral domain *λ* > 300 nm, i.e. in the range 327–470 nm. The differences between the theoretical values of absorption maxima and the experimental spectral data can arise from the fact that the calculations were carried out for gas phase, while the experimental values were obtained in solvent at room temperature.

**Table 10:** **Theoretical wavelengths and oscillator strengths computed by CI-AM1 and CI-PM3 methods for (1–4)**

Compound		***λ (nm)***	***f***	***E***_***λ***_ ***(eV)***	Assignment (Transition):
					Occ. MO --> Unocc. MO
(E)-1	AM1	300.07	1.113	4.132	HOMO → LUMO_+1_
	PM3	316.11	1.175	3.922	HOMO → LUMO_+1_
(Z)-1	AM1	312.90	0.467	3.962	HOMO → LUMO_+1_
	PM3	320.87	0.116	3.864	HOMO → LUMO; HOMO_-1_ → LUMO
(E)-2	AM1	315.55	1.507	3.929	HOMO → LUMO_+1_
	PM3	321.69	1.274	3.854	HOMO → LUMO_+1_
(Z)-2	AM1	314.17	0.770	3.946	HOMO → LUMO_+1_; HOMO → LUMO_+2_
	PM3	314.71	0.110	3.940	HOMO_-2_ → LUMO; HOMO_-7_ → LUMO
(E)-3	AM1	366.98	0.407	3.379	HOMO → LUMO_+2;_ HOMO_-4_ → LUMO_+2_
	PM3	319.86	0.641	3.876	HOMO → LUMO_+2_
(Z)-3	AM1	321.84	0.234	3.852	HOMO → LUMO_+2_
	PM3	315.85	0.085	3.925	HOMO_-1_ → LUMO; HOMO_-6_ → LUMO
(E)-4	AM1	360.24	0.281	3.442	HOMO → LUMO_+2_; HOMO_-4_ → LUMO_+2_
	PM3	300.95	0.339	4.120	HOMO → LUMO_+2_
(Z)-4	AM1	320.67	0.186	3.866	HOMO → LUMO_+2_
	PM3	317.78	0.089	3.902	HOMO_-1_ → LUMO; HOMO_-6_ → LUMO

**Table 11:** **Experimental wavelengths determined from UV–vis absorption spectra**

Compound	Solvent	$\lambda_{\text{max}}^{\text{exper}}$ (nm)
1	Chloroform	327; 470
2	Chloroform	344; 441
	Dioxane	356
	Dichloromethane	352
	Dimethylformamide	358
	Dimethyl sulfoxide	362
3	Tetrahydrofuran	344
	Toluene	345
	Dimethylformamide	345.5; 442
4	Dichloromethane	344
	Dimethylformamide	342

### Spin densities

**Figure 12:**
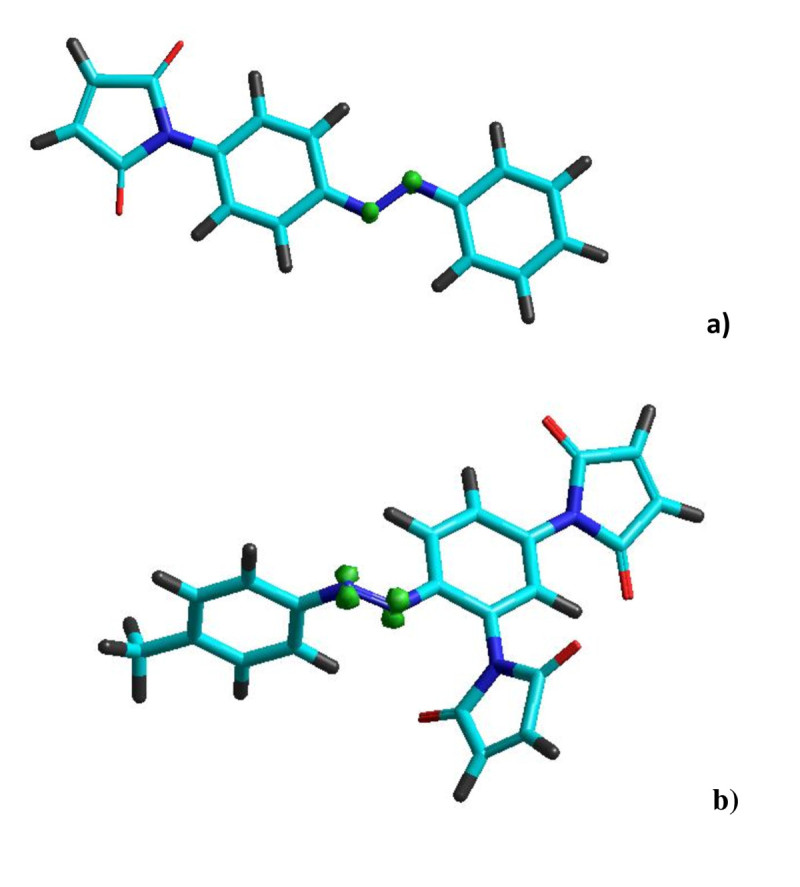
**Structure and spin density distribution (shown as green spheres) of the first excited triplet state (T**_**1**_ **) for a) (E)-1 and b) (E)-3 computed by AM1 method.**

Intersystem crossing from the excited singlet state to an intermediate triplet state may occur at some point where the potential energy surfaces of the singlet and triplet states intersect at a common geometry of the studied molecule (Morley et al. 2004). Our computational results based on semi-empirical models (AM1 and PM3) indicated that for studied azobenezene derivatives (1–4), the first triplet state (T_1_) lies around 8 to 43 kcal/mol above the ground state (S_0_). The two unpaired electrons in the triplet state may not be distributed uniformly throughout the molecular frame but rather localized at certain atomic centers (Morley et al. 2004). A visual illustration of the spin densities of the two azobenzene derivatives, (E)-1 and (E)-3, in their first excited triplet state (T_1_) is presented in Figure 12. Note that, Figure 12 shows the optimized geometries for T_1_ excited state, computed by AM1 method using unrestricted Hartree-Fock (UHF) spin pairing level. As one can see, AM1 computation results indicated that, for both compounds (E)-1 and (E)-3, the electron spin density in the first excited triplet state is concentrated at azo nitrogen atoms (Figure 12). In contrast, additional calculation at PM3 level (for both (E)-1 and (E)-3) has revealed that the electron spin density in state T_1_ is localized at nitrogen atom from maleimide group. For the compound (E)-2, both AM1 and PM3 methods have disclosed the spin density concentrated at azo nitrogen atoms. Morley and co-workers (Morley et al. 2004) suggested that a greater localization of spin density at the reactive azo nitrogen atoms in the excited triplet state may be correlated with the lower photochemical stability of some azobenzene derivatives. The experimental results showed that azo derivatives 2 and 3 provide high yields of the (Z) isomers at irradiation with UV light (365 nm) (Airinei et al. 2011b).

## Conclusions

Quantum mechanics calculations were performed to investigate the structure and stability of (E) / (Z) isomers of azobenzene derivatives 1–4 containing maleimide groups. The calculations at the theoretical level of PM3, MNDO, RHF/6-31+G(d,p) and B3LYP/6-31+G(d,p) indicated that (E) isomers of 1–4 are the most stable.

The available X-ray-structure-analysis data for the compound (E)-1, (E)-1-(4-(phenyldiazenyl)phenyl)-1H-pyrrole-2,5-dione, were used to validate the modeling geometries computed at the theoretical levels AM1, PM3, RHF/6-31+G(d,p) and B3LYP/6-31+G(d,p). The results revealed that ab-initio calculations at RHF/6-31+G(d,p) level yield the most accurate prediction on (E)-1 structure, giving a deviation error from crystallographic data of about 5.00% for bond lengths and 0.97% for valence angles.

For all azobenzenes studied in this paper, the applied semi-empirical methods (AM1 and PM3) have indicated that the calculated net atomic charges of azo nitrogen atoms (N=N) are smaller in case of (E) isomers comparing with (Z) isomers. The computed values of the energy gap of studied azobenzene derivatives (1–4), in their ground state, varied from 7.022 eV to 7.860 eV. In most of the cases, the calculated values of energy gap, electronegativity and chemical hardness were slightly higher for (Z) isomers than for (E) isomers. The plotting of frontier molecular orbitals (HOMO and LUMO) for 1–4 showed that the HOMO levels were spread mainly over aromatic rings and azo group. In contrast, the LUMO levels were almost uniformly distributed over maleimide moiety. The theoretical electronic spectra of azobenzene derivatives 1–4 were computed by configuration-interaction method (CI) at the level of semi-empirical Hamiltonians, revealing the transitions and the corresponding oscillator strengths.

## Nomenclature

AM1 Austin Method 1 (semi-empirical model)

*ARE* average relative error

B3LYP Becke exchange and Lee-Yang-Parr correlation functionals

CI configuration interaction

DFT density functional theory

*DM* dipole moment

(E) isomer notation (*Entgegen*): higher-priority substituents are on the opposite side

*E* binding energy

*EA* electron affinity

*E*_*λ*_ excitation energy

*E*_*H*_ hydration energy

*E*_*rel*_ relative strain energy

Δ*E* energy gap

ESP electrostatic potential

HOMO highest occupied molecular orbital

*f* oscillator strength

*H* heat of formation

*IP* ionization potential

LUMO lowest unoccupied molecular orbital

MNDO Modified Neglect of the Diatomic Overlap (semi-empirical model)

MO molecular orbital

*n* lone pairs electrons

*N* number of data (available experimental data)

OMO occupied molecular orbital

PM3 Parameterization Method 3 (semi-empirical model)

QM quantum mechanics

QSAR quantitative structure–activity relationship

RHF restricted Hartree-Fock basis

*RMSD* root-mean-square error deviation

SCF self-consistent field method

S_0_ ground state

T_1_ first excited triplet state

UHF unrestricted Hartree-Fock basis

UMO unoccupied (virtual) molecular orbital

*X* structural parameter

(Z) isomer notation (*Zusammen*): higher-priority substituents are on the same side

**Greeks letters**

*χ* electronegativity

ϵ energy (eigenvalue)

*η* chemical hardness

*λ* wavelength

*μ* chemical potential

π bonding molecular orbital (π-symmetry)

π* antibonding molecular orbital (π-symmetry)

*ω* electrophilicity index

Ψ wave-function (orbital)

**Subscripts and superscripts**

*calc* calculated (theoretical) value

*exp* experimental value

*i* positive integer number (iteration index)

*j* positive integer number (iteration index)

*max* maximum

## Authors’ original submitted files for images

Below are the links to the authors’ original submitted files for images. Authors’ original file for figure 1 Authors’ original file for figure 2 Authors’ original file for figure 3 Authors’ original file for figure 4 Authors’ original file for figure 5 Authors’ original file for figure 6 Authors’ original file for figure 7 Authors’ original file for figure 8 Authors’ original file for figure 9 Authors’ original file for figure 10 Authors’ original file for figure 11 Authors’ original file for figure 12
