# Supplementary figures and images for: Molecular structure and modeling studies of azobenzene derivatives containing maleimide groups
Source: Springerplus. 2013 Oct 31;2(1):586. doi: 10.1186/2193-1801-2-586 (PMC3825101; doi:10.1186/2193-1801-2-586)

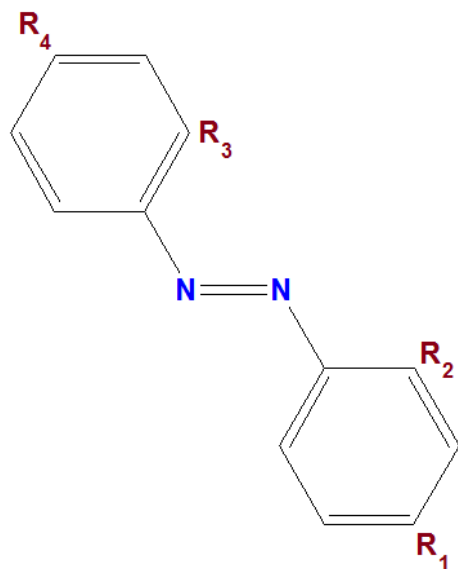

(E)

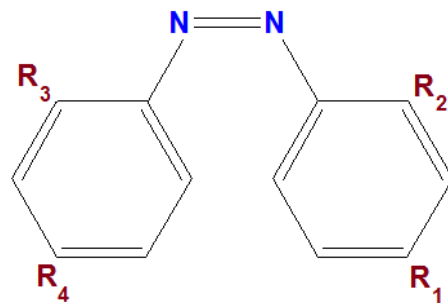

(Z)

| Compound | R <sub>1</sub>   | R <sub>2</sub>   | R <sub>3</sub> | R <sub>4</sub> |
|----------|------------------|------------------|----------------|----------------|
| 1        |                  | -H               | -H             | -H             |
| 2        | Ph-CO-NH-<br>    | -H               | -H             | -H             |
| 3        | -CH <sub>3</sub> | -H               |                |                |
| 4        | -H               | -CH <sub>3</sub> |                |                |

Supplement: Supplementary file 1 — Authors’ original file for figure 1 [file 40064_2013_637_MOESM1_ESM.pdf]

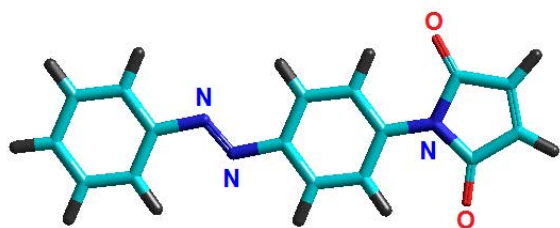

(E)-1

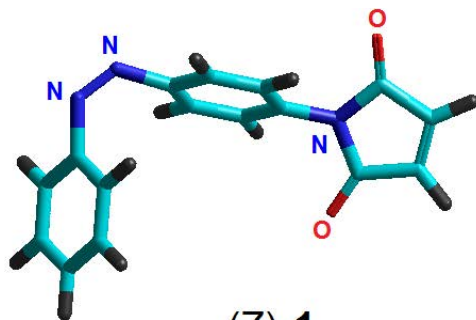

(Z)-1

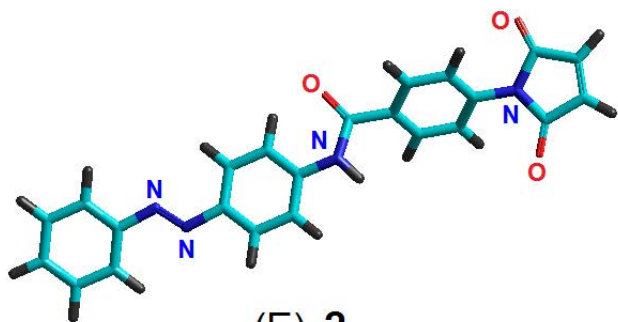

(E)-2

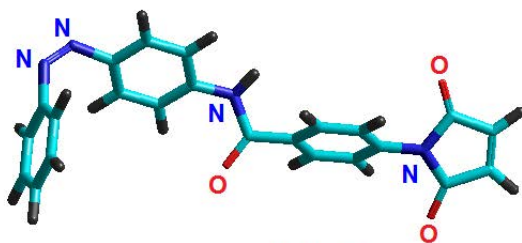

(Z)-2

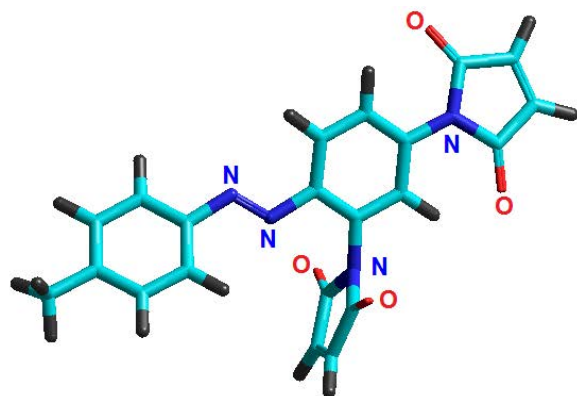

(E)-3

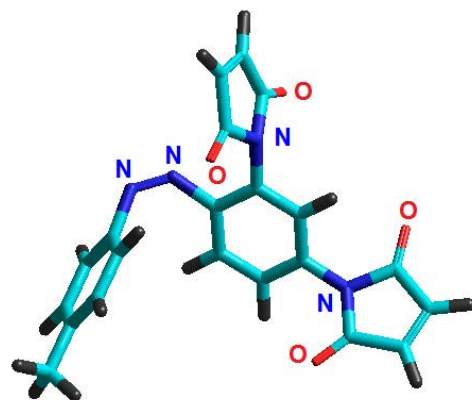

(Z)-3

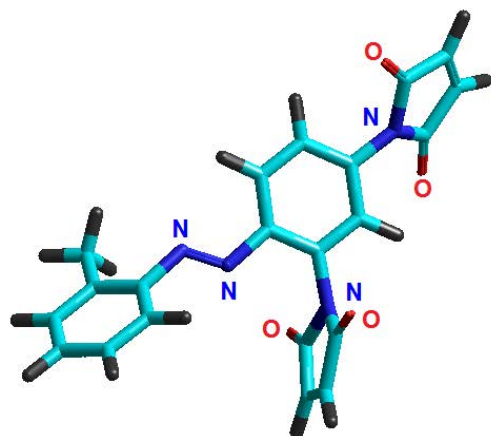

(E)-4

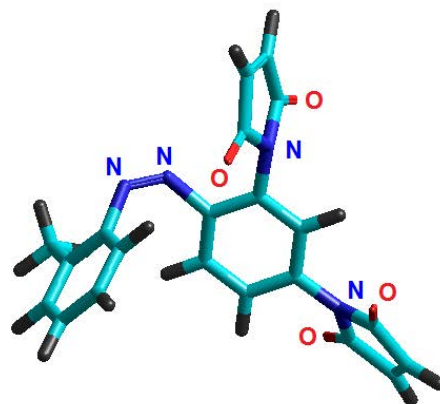

(Z)-4

Supplement: Supplementary file 2 — Authors’ original file for figure 2 [file 40064_2013_637_MOESM2_ESM.pdf]

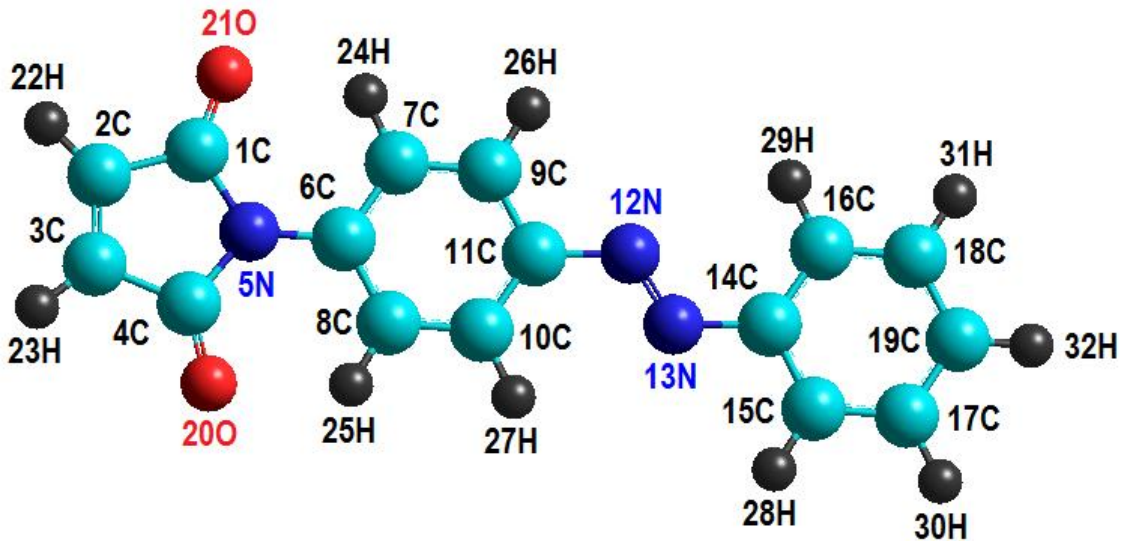

Supplement: Supplementary file 3 — Authors’ original file for figure 3 [file 40064_2013_637_MOESM3_ESM.pdf]

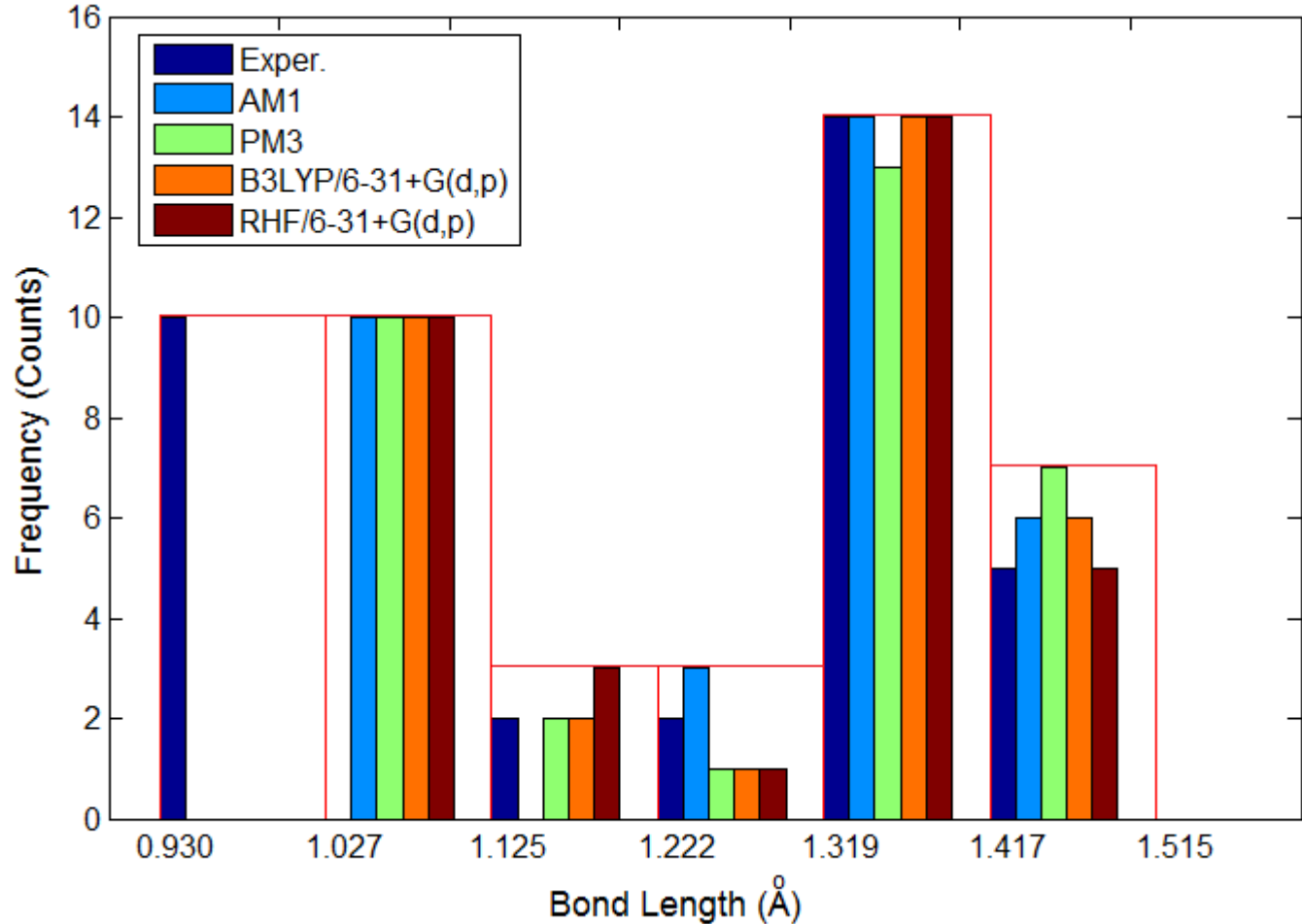

Supplement: Supplementary file 4 — Authors’ original file for figure 4 [file 40064_2013_637_MOESM4_ESM.pdf]

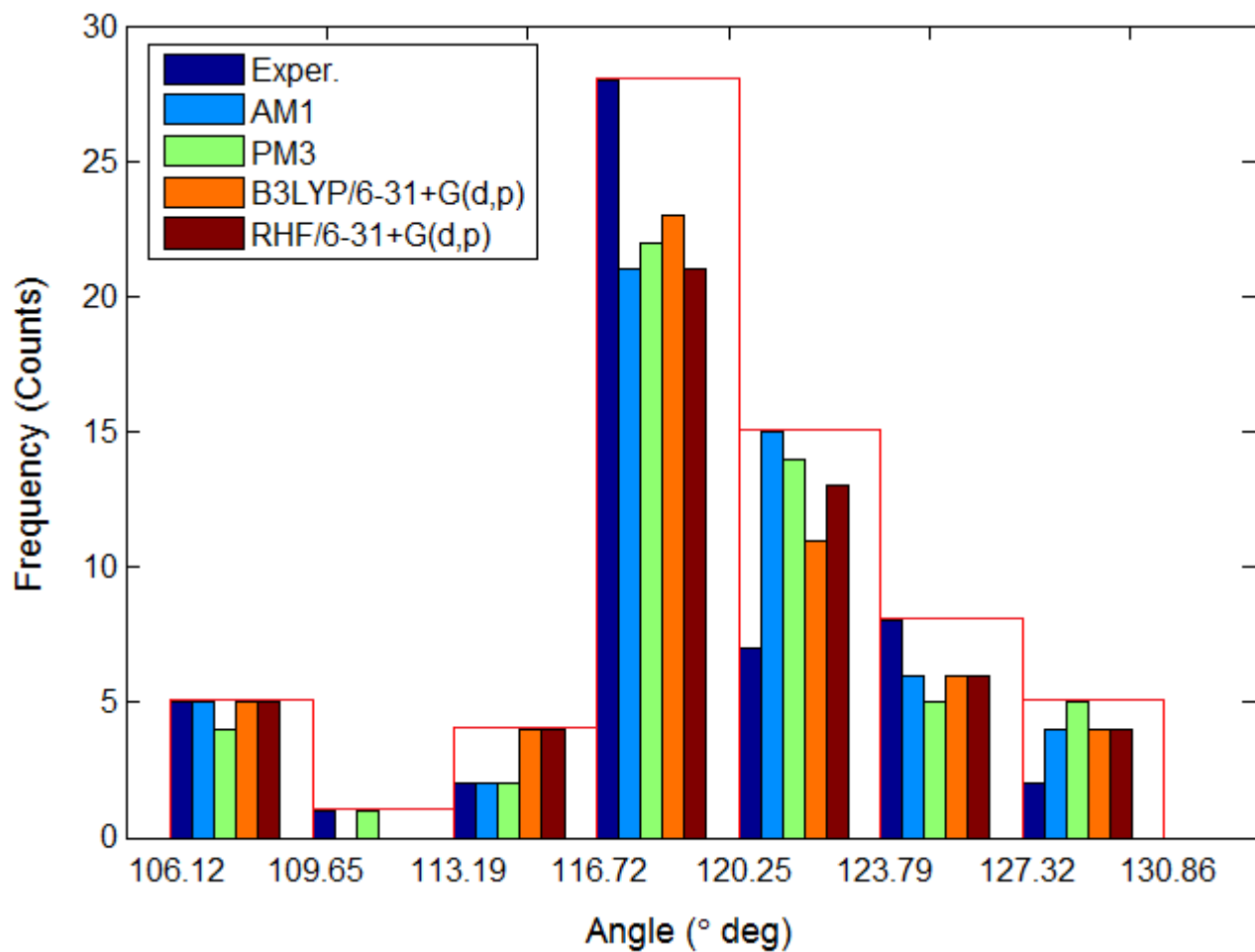

Supplement: Supplementary file 5 — Authors’ original file for figure 5 [file 40064_2013_637_MOESM5_ESM.pdf]

**(a)**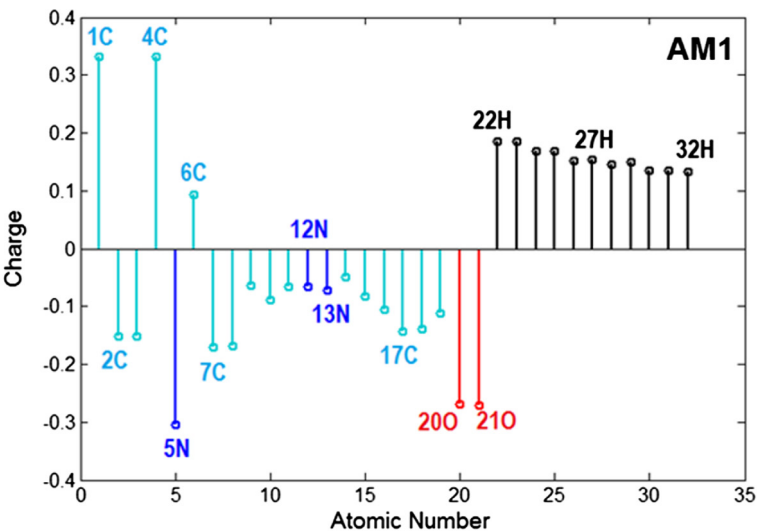**(b)**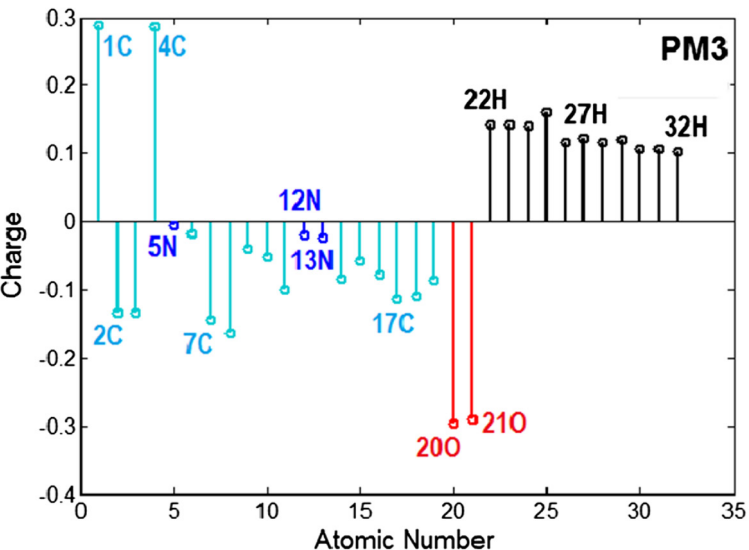

Supplement: Supplementary file 6 — Authors’ original file for figure 6 [file 40064_2013_637_MOESM6_ESM.pdf]

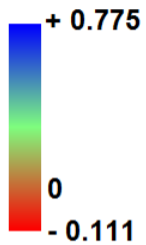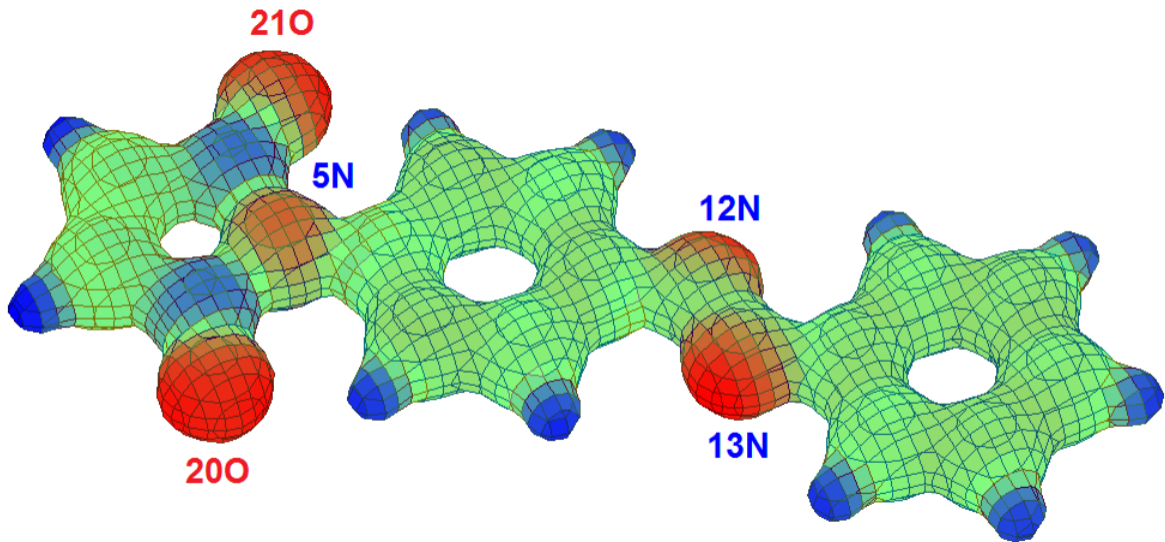

Supplement: Supplementary file 7 — Authors’ original file for figure 7 [file 40064_2013_637_MOESM7_ESM.pdf]

Energy (eV)

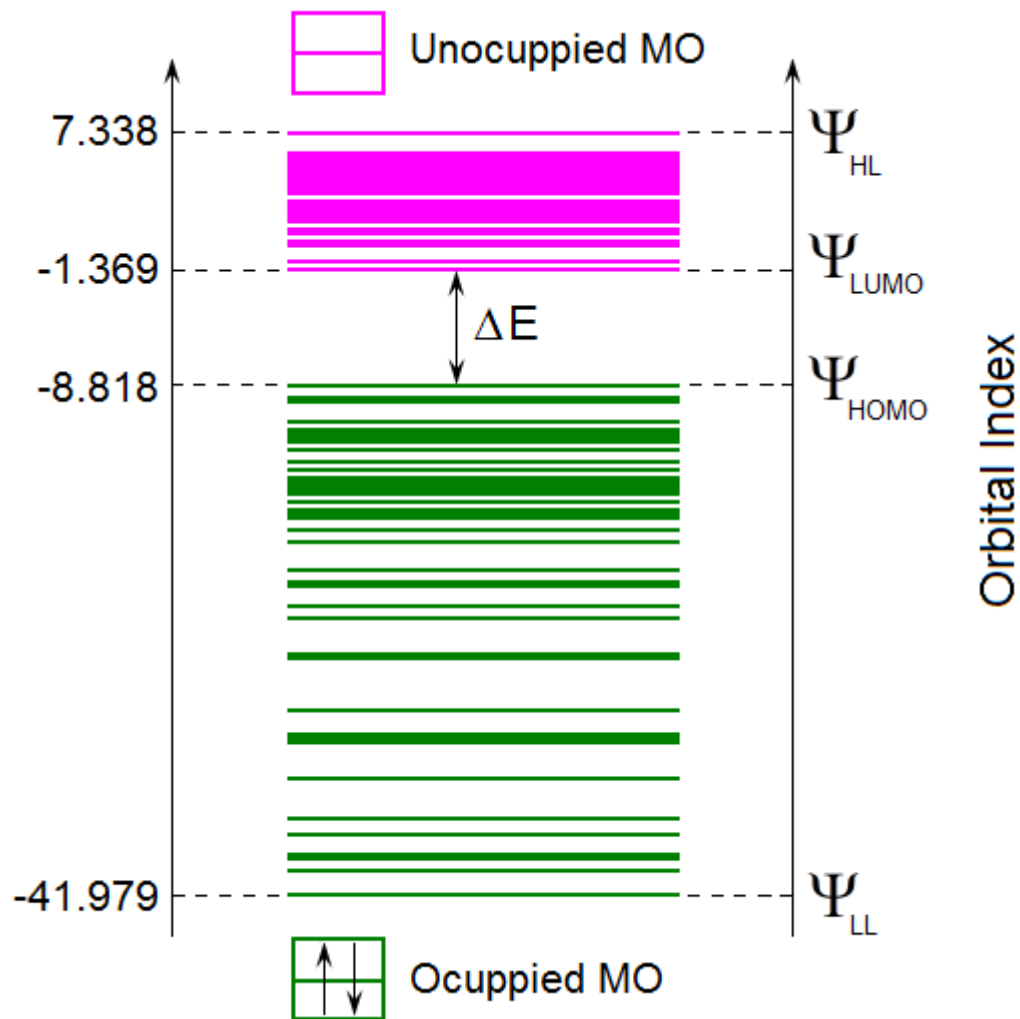

Supplement: Supplementary file 8 — Authors’ original file for figure 8 [file 40064_2013_637_MOESM8_ESM.pdf]

(E)-1: HOMO

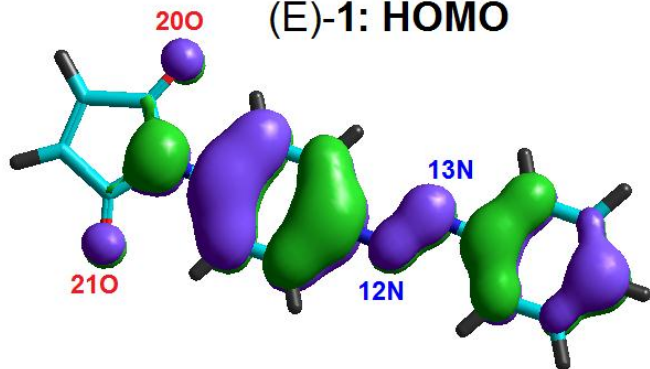

(E)-1: LUMO

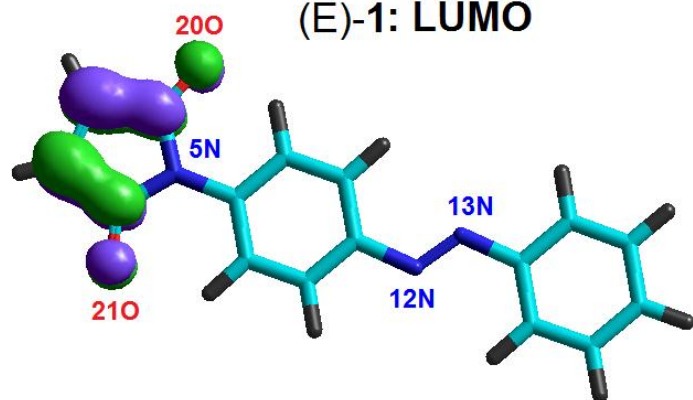

(Z)-1: HOMO

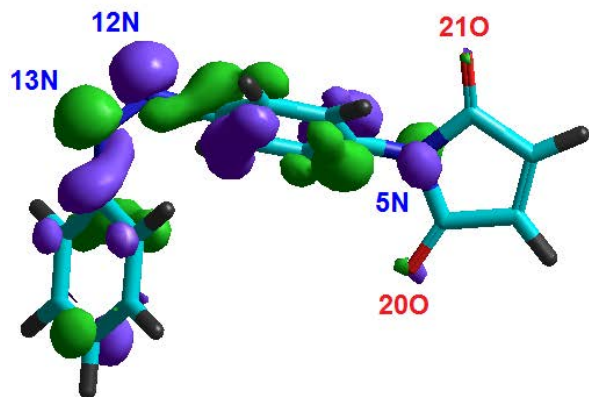

(Z)-1: LUMO

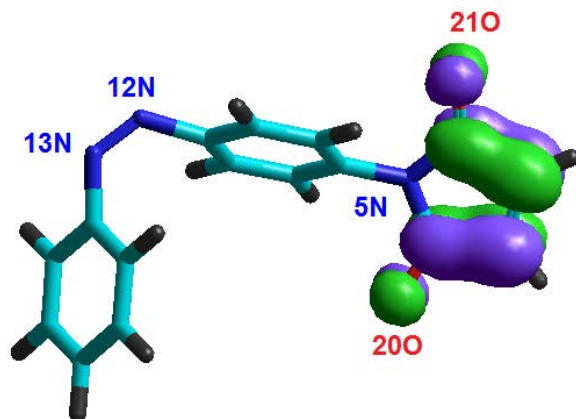

Supplement: Supplementary file 9 — Authors’ original file for figure 9 [file 40064_2013_637_MOESM9_ESM.pdf]

**(a)**

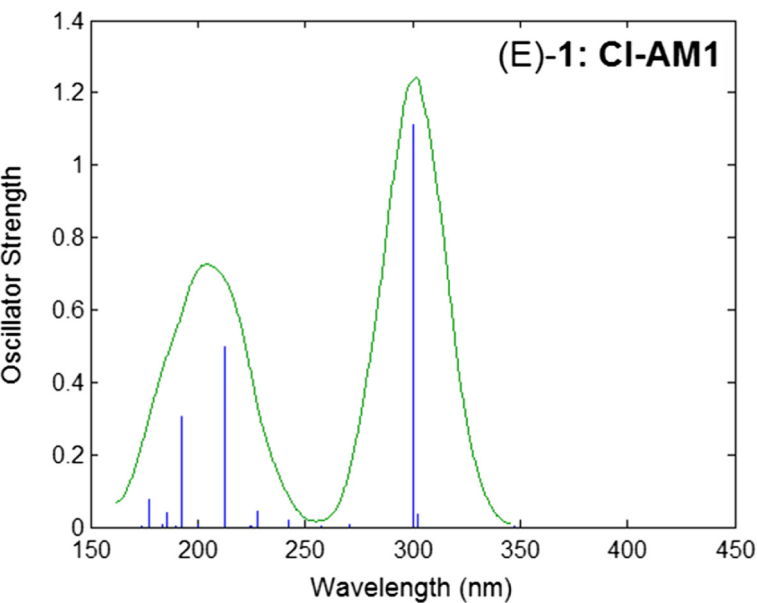

**(b)**

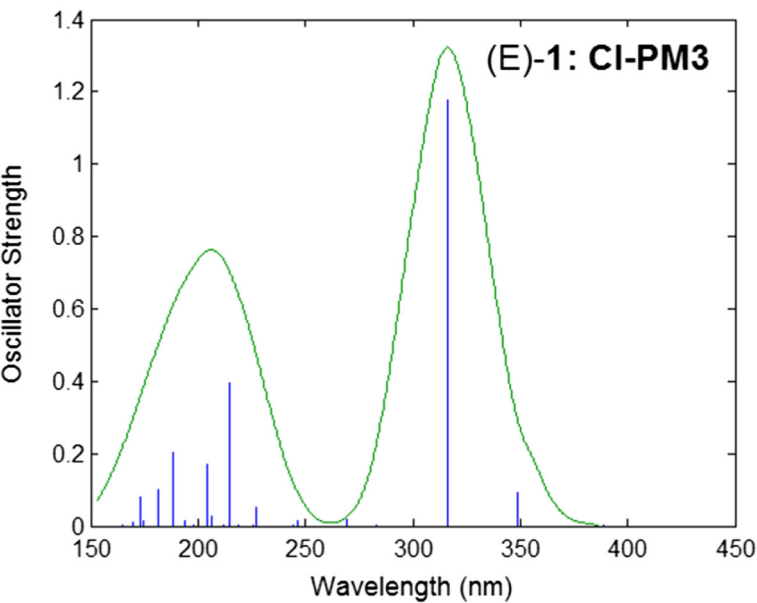

Supplement: Supplementary file 10 — Authors’ original file for figure 10 [file 40064_2013_637_MOESM10_ESM.pdf]

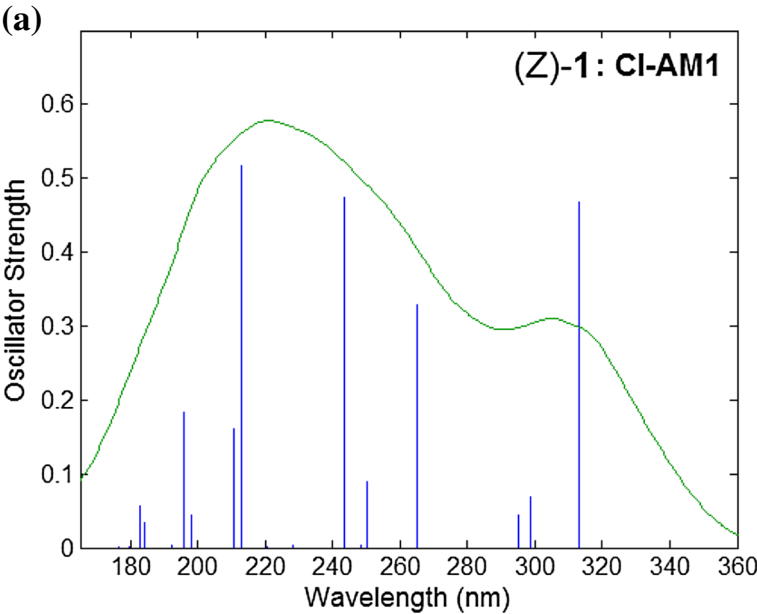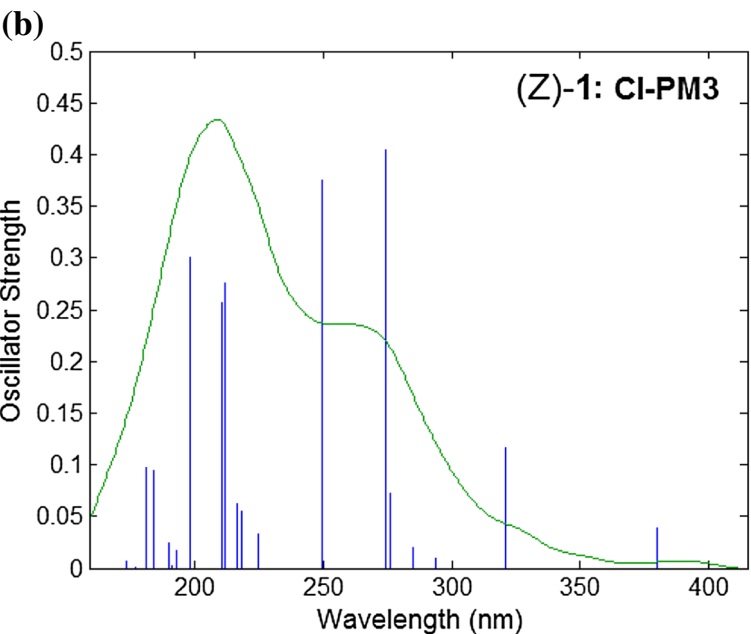

Supplement: Supplementary file 11 — Authors’ original file for figure 11 [file 40064_2013_637_MOESM11_ESM.pdf]

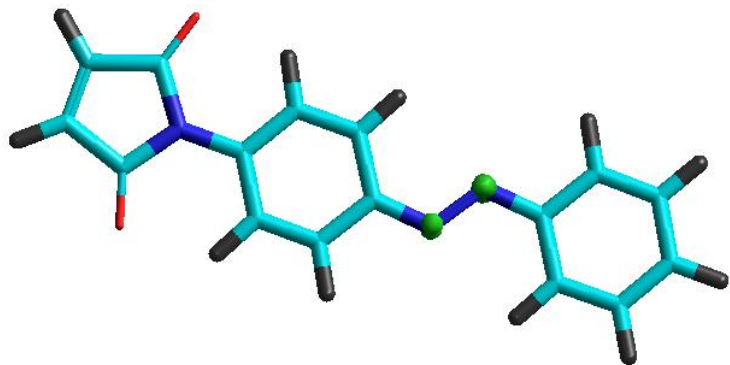

a)

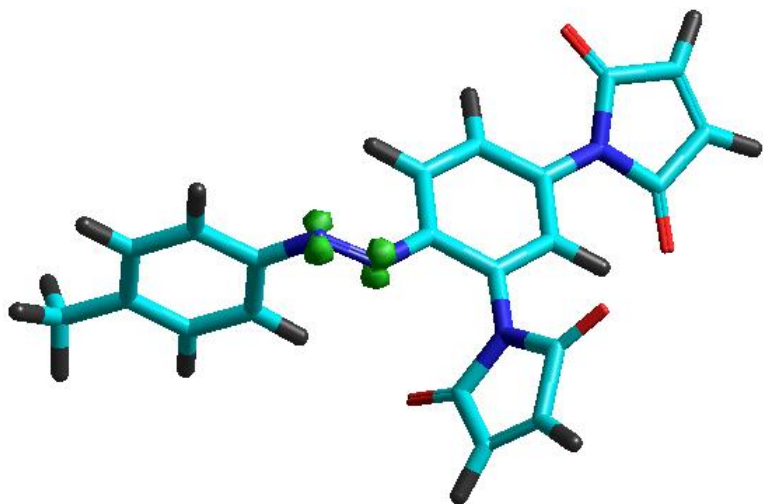

b)

Supplement: Supplementary file 12 — Authors’ original file for figure 12 [file 40064_2013_637_MOESM12_ESM.pdf]
